# The role of heterotrophic plasticity in coral response to natural low‐light environments

**DOI:** 10.1002/ece3.70278

**Published:** 2024-09-23

**Authors:** Yong Luo, Xiaolei Yu, Lintao Huang, Jianfeng Gan, Xinming Lei, Lei Jiang, Chengyue Liu, Youfang Sun, Meng Cheng, Yuyang Zhang, Guowei Zhou, Sheng Liu, Jiansheng Lian, Hui Huang

**Affiliations:** ^1^ CAS Key Laboratory of Tropical Marine bio‐Resources and Ecology, Guangdong Provincial Key Laboratory of Applied Marine Biology South China Sea Institute of Oceanology (SCSIO), Chinese Academy of Sciences Guangzhou China; ^2^ CAS‐HKUST Sanya Joint Laboratory of Marine Science Research, Key Laboratory of Tropical Marine Biotechnology of Hainan Province Sanya Institute of Ocean Eco‐Environmental Engineering, SCSIO Sanya China; ^3^ Sanya National Marine Ecosystem Research Station, Tropical Marine Biological Research Station in Hainan Chinese Academy of Sciences Sanya China; ^4^ University of Chinese Academy of Sciences Beijing China; ^5^ South China Institute of Environmental Sciences Ministry of Ecology and Environment of the People's Republic of China Guangzhou China; ^6^ Southern Marine Science and Engineering Guangdong Laboratory (Guangzhou) Guangzhou China; ^7^ Innovation Academy of South China Sea Ecology and Environmental Engineering Chinese Academy of Sciences Guangzhou China

**Keywords:** coastal darkening, coral nutrition, fringing reefs, *Galaxea fascicularis*, stable isotopes

## Abstract

Coastal darkening is emerging as a global threat to fringing reefs. While some reef‐building corals exhibit resistance to low‐light environments, the mechanisms behind this resistance, particularly the role of coral hosts, remain inadequately understood. Here, we investigated variations in underwater photosynthetically active radiation (PAR) and employed the Bayesian stable isotope mixing model (MixSIAR) to estimate the contributions of autotrophic (i.e., dissolved inorganic matter, DIM) and heterotrophic sources (i.e., particulate organic matter, POM, and dissolved organic matter, DOM) to the nutrition of the reef coral *Galaxea fascicularis* on the Luhuitou turbid reef in the northern South China Sea. Our findings revealed that the heterotrophic contribution to coral nutrition increased to 58.5% with decreasing PAR and that the heterotrophic contribution was significantly negatively correlated with δ^13^C difference between host and symbiont (δ^13^C_h–s_). Moreover, we observed significant seasonal variations in the respective contributions of POM and DOM to coral nutrition, linked to the sources of these nutrients, demonstrating that *G. fascicularis* can selectively ingest POM and DOM based on their bioavailability to enhance its heterotrophic contribution. This heterotrophic plasticity improved the low‐light resistance of *G. fascicularis* and contributed to its prominence within coral communities. However, with a low‐light threshold of approximately 3.73% of the surface PAR for *G. fascicularis*, our results underscore the need for effective strategies to mitigate low‐light conditions on nearshore turbid reefs. In summary, our study highlights the critical role of heterotrophic plasticity in coral responses to natural low‐light environments, suggesting that some reef‐building corals with such plasticity could become dominant or resilient species in the context of coastal darkening.

## Graphical abstract

Low‐light environments are driving vertical reef compression and favoring depth generalists within coral communities lying on inshore turbid reefs, highlighting an emerging local threat that is affecting fringing reefs on a global scale. Our study provides direct field evidence that the reef coral *Galaxea fascicularis* exhibits heterotrophic plasticity, selectively utilizing heterotrophic sources based on their bioavailability, which in turn enhances its heterotrophic contribution, bolstering its resistance to low‐light conditions. These findings suggest that reef‐building corals with such heterotrophic plasticity may have a greater potential to persist or even dominate in environments affected by coastal darkening.

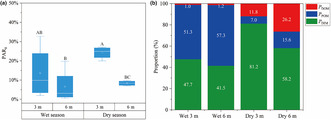

## 1 INTRODUCTION

In recent decades, the global transportation of terrestrial‐derived materials from catchments to coastal waters has significantly increased, driven by escalating anthropogenic disturbances and regional climate changes (Magris et al., 2019; Syvitski et al., 2005). This trend, coupled with the resuspension of surface fine‐grained sediments due to wave and tidal action (Bainbridge et al., 2018) and the proliferation of phytoplankton blooms induced by nutrient enrichment (Hayashida et al., 2020), has led to a substantial rise in suspended solids in the water column (Zweifler et al., 2021). Given the significant light attenuation properties of suspended solids, this results in a long‐term decrease in the transparency of coastal waters, a phenomenon termed “coastal darkening” (Aksnes et al., 2009; Cacciapaglia & van Woesik, 2016). For instance, sediment accumulation rates in Singapore have increased from less than 6 mg cm^−2^ d^−1^ in the 1970s (Chan, 1980) to at least 9.8 mg cm^−2^ d^−1^ at present, attributed to coastal development (Morgan et al., 2020). This rapid light attenuation has resulted in a shallow euphotic depth of less than 11 m (Morgan et al., 2020). Coastal darkening exerts a profound influence on the growth and distribution of benthic organisms that depend on photosynthetically active radiation (PAR) for photosynthesis (Canto et al., 2021), including macroalgae (Blain et al., 2021), seagrass (Yamamoto et al., 2019), and reef‐building corals (Chow et al., 2019). Among these organisms, reef‐building corals have garnered significant attention due to their role in forming the fundamental framework of coral reef ecosystems (Magris et al., 2019; Zweifler et al., 2021). The reduction in underwater PAR significantly diminishes the energy and nutrients supplied to corals by their algal endosymbionts (Iluz & Dubinsky, 2015), often leading to coral bleaching or mortality (Jones et al., 2020; Strahl et al., 2019). These adverse effects jeopardize the vital ecosystem services provided by coral reefs, including food provisioning, livelihood opportunities, carbon sequestration, and storm protection (Eddy et al., 2021; Woodhead et al., 2019). It is evident that coastal darkening caused by increased suspended solids is emerging as a global threat to fringing reefs.

Notably, certain coral species characterized by massive or encrusting growth forms exhibit robust growth even under prolonged or frequent exposure to sub‐optimal light conditions (Browne et al., 2012; Chow et al., 2019; Luter et al., 2021; Zweifler et al., 2021). This is evidenced by the fact that corals on most turbid reefs globally are dominated by massive or encrusting growth types, characterized by high cover and low species diversity (Loiola et al., 2019; Morgan et al., 2017). Reef‐building corals possess the ecologically important trait of being mixotrophic (Conti‐Jerpe et al., 2020; Eddy et al., 2021), that is, obtaining energy and nutrients (e.g., carbohydrates, lipids, minerals, proteins, and trace elements) for their physiological metabolism both autotrophically (through the absorption of photosynthetic products from their symbiotic algae) and heterotrophically (by feeding on particulate organic matter (POM) and dissolved organic matter (DOM) in the reef water column) (Houlbreque & Ferrier‐Pages, 2009; Iluz & Dubinsky, 2015). These findings suggest that the tolerance of reef‐building corals to low‐light environments on turbid reefs may be related to their trophic plasticity, particularly increased heterotrophic nutrition (Anthony & Fabricius, 2000; Radice et al., 2019; Sturaro et al., 2021). For instance, heterotrophic nutrition can meet 15%–35% of the daily metabolic requirements of healthy corals and up to 100% for bleached corals (Houlbreque & Ferrier‐Pages, 2009). In recent years, heterotrophic plasticity has received attention for its role in enhancing coral resistance to global warming‐induced bleaching events (Conti‐Jerpe et al., 2020; Grottoli et al., 2006). However, the role of heterotrophic plasticity in coral responses to low‐light environments remains unclear, particularly regarding the respective contributions of POM and DOM to specific reef coral species across a light‐availability gradient in the field.

In recent decades, the natural abundance of stable isotopes of carbon (δ^13^C) and nitrogen (δ^15^N) in coral hosts and endosymbionts has been progressively utilized to broadly estimate the proportion of photoautotrophic and heterotrophic contributions to coral tissues under natural and experimental conditions (Bierwagen et al., 2018; Grottoli et al., 2006; Muscatine et al., 1989; Sturaro et al., 2021). Trophic strategies of corals have also been identified using stable isotopes in various regions, including Hong Kong (Conti‐Jerpe et al., 2020), the South China Sea (Xu et al., 2020), the Maldives (Radice et al., 2019), the south and north regions of Taiwan Island (Sturaro et al., 2021), and the central Pacific Islands (Fox et al., 2018), demonstrating that coral trophic strategies of corals vary with natural environments. However, fewer studies have quantitatively assessed the contribution of different nutrient sources to coral nutrition compared to qualitative estimates (Price et al., 2021). In recent years, Bayesian Stable Isotope Mixing Modeling (MixSIAR), an emerging ecological research technique (Stock et al., 2018), has been increasingly applied to assess nutrient sources and their contributions to marine animals (Cutajar et al., 2022; García‐Seoane et al., 2023). For example, Price et al. (2021) utilized the MixSIAR model to evaluate the proportional contributions of dissolved inorganic matter (DIM), POM, and zooplankton to the nutrition of seven Hawaiian coral species collected from six sites around the island of O'ahu, Hawaii.

Luhuitou fringing reef, located southeast of Sanya Bay (18°12′ N, 109°28′ E), is one of the best‐developed fringing reefs in the northern South China Sea (Zhang, 2001). Coral cover on the Luhuitou fringing reef has dramatically declined in recent decades (Hughes et al., 2013). Zhao MeiXia et al. (2012) attributed this decline may be due to overfishing, destructive fishing practices, reef dredging, mariculture, and tourism activities. However, Li et al. (2013) reported that the average sediment accumulation rate from 2007 to 2009 was nearly 20 mg cm^−2^ d^−1^ and found a strong negative correlation between silt‐clay‐sized sediment accumulation rates and coral cover. A recent study by Luo et al. (2022) demonstrated that rapid light attenuation over the short depth range on the Luhuitou fringing reef, caused by elevated suspended sediment concentrations, resulted in a shallow euphotic depth (<11 m). This condition compressed the vertical distribution of coral growth and affected coral assemblages. These results suggest that increased suspended sediment concentrations and low light conditions are significant threats to the Luhuitou fringing reef. The reef coral *Galaxea fascicularis* (Linnaeus, 1767), which is widely distributed across inshore fringing reefs in the Indo‐Pacific region (Veron, 2000), and is an ecologically important and dominant species on the Luhuitou fringing reef (Luo et al., 2022). Due to its resistance to environmental changes and stresses (Yu et al., 2024; Zhu et al., 2022), it serves as a valuable model organism for studying the response mechanism of coral hosts to low‐light environments. In addition, POM, a heterotrophic source for corals, exhibits significant seasonal variations in its sources and components (Cao et al., 2017; Luo et al., 2022). Therefore, the Luhuitou fringing reef provides an excellent field site for exploring the role of heterotrophic plasticity in the resistance of specific coral species to low‐light environments in the context of coastal darkening.

In this study, we propose the hypothesis that in low‐light environments, the reef coral *G. fascicularis* may selectively utilize heterotrophic sources (POM and DOM) based on their bioavailability to enhance their heterotrophic contribution. To investigate this, we examined variations in PAR and estimated the respective contributions of DIM, POM, and DOM to *G. fascicularis* using the MixSIAR model (Price et al., 2021; Stock et al., 2018) across a light gradient on the Luhuitou fringing reef, Sanya, China. Through this study, we aim to provide direct evidence that some reef‐building corals can exhibit heterotrophic plasticity across environmental gradients at small spatial scales.

## 2 MATERIALS AND METHODS

### 2.1 Environmental data collected by shipboard surveys

**FIGURE 1:**
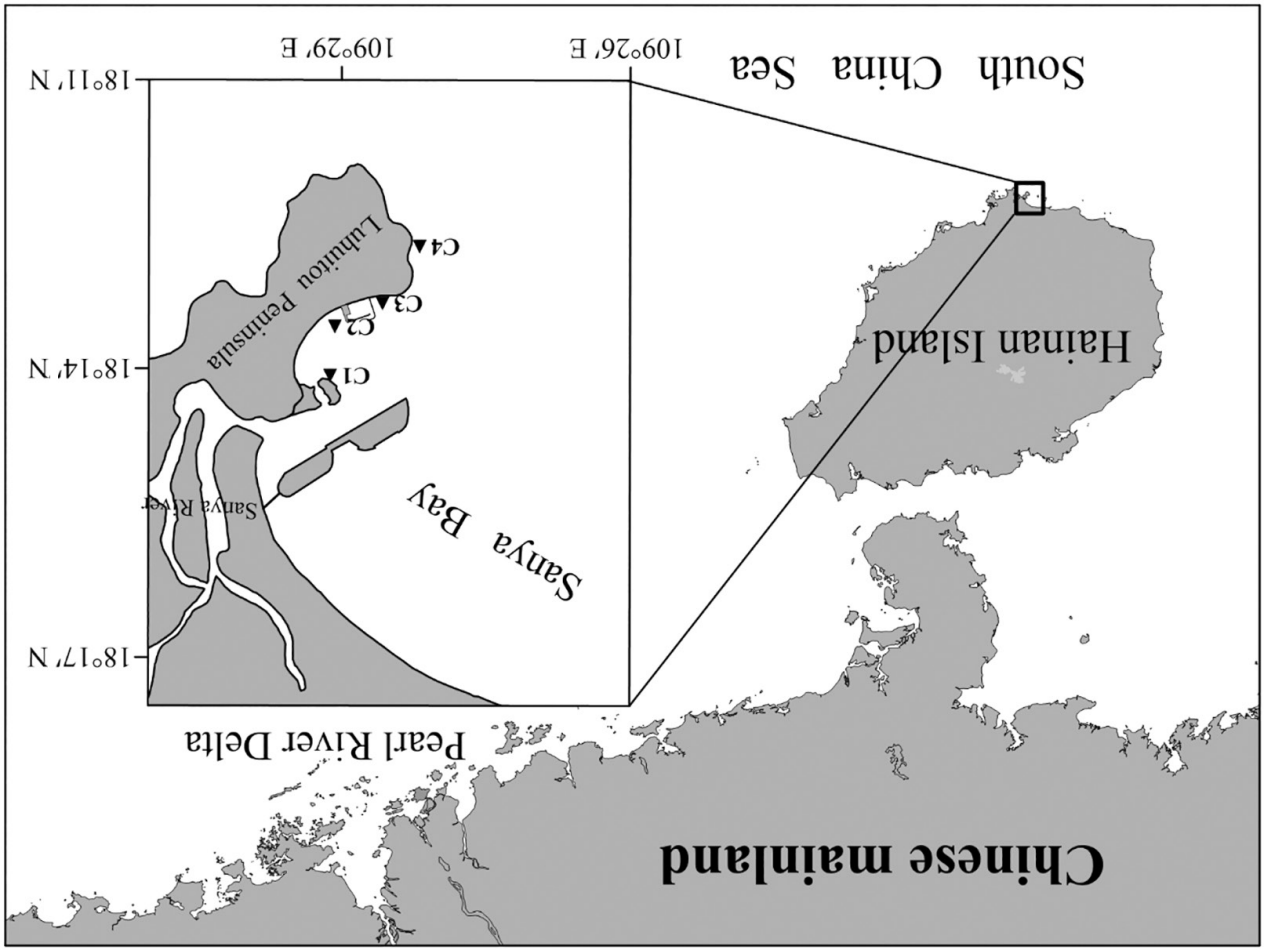
Map of sampling sites (C1–C4) (black solid triangles) on the Luhuitou fringing reef located in Sanya Bay, northern South China Sea.

This study was conducted on the Luhuitou fringing reef, located along the west coast of the Luhuitou Peninsula in southeast Sanya Bay, Hainan Island, northern South China Sea (Figure 1). Surface (approximately 0.5 m below the water surface) and bottom (approximately 1.0 m from the seafloor) seawater samples were collected in triplicate at sites C1–C4 using a 5 L Niskin water sampler (General Oceanics, Miami, FL, USA) during two cruises on August 24, 2019 (wet season) and May 3, 2020 (dry season). Underwater PAR data for both wet and dry seasons at sites C1–C4 were collected using a Li‐COR 1500 data logger (LI‐COR, Lincoln, NE, USA) equipped with a manufacturer‐calibrated Li‐COR LI‐193SA underwater quantum sensor (“PAR” sensor) following the methods described in Luo et al. (2022). Briefly, the PAR sensor was attached by cable to a LI‐COR Lowering Frame with two lead blocks attached. After determining the water depth at the site, the PAR sensor was allowed to fall freely to a depth of approximately 3~5 cm below the water surface using Prompt On Log mode to record valid data (surface PAR data, PAR_0_) at 1 Hz for 30~60 s. Following this, the PAR sensor was rapidly lowered to a water depth of 0.5 m, and this process was repeated at 0.5 m intervals until reaching the seafloor. The site‐specific diffuse attenuation coefficient of the PAR (*K*_d‐PAR_) was then calculated using a modified Beer–Lambert equation for light attenuation, as described in Morgan et al. (2020). A calibrated conductivity‐temperature‐depth probe (Ocean Seven 304 Plus, Idronaut, Italy) was used for the in situ measurements of seawater temperature (T) and salinity (S). Suspended solids concentration (SSC) was determined by filtering 1 L water samples through weighed standard filter paper (47 mm, nominal pore size 0.45 μm). The residue was dried in an oven at 60°C to a constant weight (Faisal et al., 2022). Chlorophyll a (Chl‐a) in seawater samples was filtered through glass fiber filters (25 mm, nominal pore size 0.7 μm) and extracted in the dark for 24 h at 4°C with 90% acetone (Browne et al., 2015). Measurements of Chl‐a were performed using a fluorometer (Turner Designs, CA, USA), with calculations following the method described by Jeffrey and Humphrey (1975).

### 2.2 Coral surveys and sampling

At sites C1–C4, two 50 m transects were conducted at depths of 3 and 6 m, parallel to the shoreline, to assess live coral cover through video surveys during the late wet season (October 24, 2019). Corals were identified to the species level, and the cover of *G. fascicularis* as well as its percentage of the total coral cover was calculated. Since the field surveys were conducted under favorable light conditions, which were largely representative of the seasonal light environment, we estimated the maximum depth (MD) of the *G. fascicularis* distribution for both the dry and wet seasons. This estimation was based on the species' low‐light threshold, using the *K*_d‐PAR_ and surface PAR (PAR_0_), as described in Equation (1).

(1) $$\mathrm{PAR}\left(\mathrm{MD}\right)={\mathrm{PAR}}_{0}\times \exp\left(-K_{d‐\mathrm{PAR}}\times \mathrm{MD}\right)$$

For further investigations, colony fragments of *G. fascicularis* (approximately 15 cm^2^) were randomly collected in triplicate at depths of 3 and 6 m using a hammer and chisel during two water sampling cruises. It should be noted that no corals were found at 6 m in site C2, so no target coral samples were taken from this location. To minimize the risk of sampling clones, the distance between each sampling fragment was maintained at greater than 5 m (Baums et al., 2019). Each collected coral fragment was immediately placed in a bag filled with seawater from the vicinity of the colony for live transport to the Tropical Marine Biological Research Station in Hainan (TMBRS), located near the Luhuitou fringing reef. Following transport, which took approximately 30 min, each fragment was snap‐frozen in liquid nitrogen and stored at −80°C for further analysis.

### 2.3 Isotopic analysis of coral samples

The methods for processing and isolating coral host tissue and algal endosymbiont for isotopic analyses were adapted from Sturaro et al. (2020). Initially, a small subsample (approximately 5 cm^2^) was taken from each collected coral fragment using a hammer and sterile chisel. The bulk coral tissue was then carefully removed from the skeleton by airbrushing with filtered seawater (0.2 μm). The resulting slurry was homogenized using an HR‐6B laboratory homogenizer/dispersion machine (Shanghai Huxi Industrial Co., Ltd., Shanghai, China).

The homogenate was then centrifuged at 2000 *g* (Eppendorf Centrifuge 5804R, Taufkirchen, Germany) for 10 min at 4°C to separate it into the animal host and endosymbiotic algal fractions. The supernatant, containing the animal host fraction, underwent an additional round of centrifugation under the same conditions to eliminate any residual algal fraction until no algal cells were detectable under the microscope. The algal pellets obtained from the initial centrifugation were washed, and resuspended in approximately 5 mL Milli‐Q water (RephiLe Bioscience, Shanghai, China), followed by centrifugation for 2 min at 90 *g* at 4°C. This washing step was repeated 10 to 12 times until nearly all animal constituents (e.g., nematocysts) and mucus were removed, as confirmed by microscopic examination. The purified algal pellets were then resuspended in centrifuge tubes with 3 mL of Milli‐Q water and thoroughly mixed using thoroughly on a homogenizer (Vortex‐2, Shanghai Husi Industrial Co., Ltd., Shanghai, China). The resuspension containing algae and the supernatant containing animal tissues were separately filtered through pre‐combusted (450°C for 6 h) GF/F glass fiber filters (pore size 0.7 μm, diameter 25 mm, Whatman, UK) under low pressure using a vacuum pump. Both fractions were subsequently acidified with 1 N HCl to remove carbonates. The prepared samples were then freeze‐dried (Biosafer‐18A, Jiangsu, China) in preparation for C and N isotope analysis. Stable isotope ratios of C and N were determined using a Sercon Integra2 elemental analyzer‐stable isotope ratio mass spectrometer (EA‐IRMS) (Sercon Ltd, Cheshire, UK) at the Third Institute of Oceanography (TIO), Ministry of Natural Resources, Xiamen, China.

The carbon isotopic signature of the animal host (δ^13^C_h_) and algal endosymbiont (δ^13^C_s_) were reported as per mil deviations of the stable isotopes ^13^C:^12^C relative to the Vienna Peedee Belemnite Limestone (v‐PDB) standard. The nitrogen isotopic signature of the animal host (δ^15^N_h_) and algal endosymbiont (δ^15^N_s_) were reported as per mil deviations of the stable isotopes ^15^N:^14^N relative to atmospheric nitrogen. The standard deviations for repeated measurements were ±0.2‰ for δ^13^C and ±0.25‰ for δ^15^N. Isotope values were calibrated using laboratory acetanilide standards (δ^13^C = −26.85 ± 0.2‰, δ^15^N = −4.21 ± 0.03‰). The elemental data for both the animal host and algal endosymbiont were presented as the ratio of carbon to nitrogen concentrations (C/N mass ratios), measured relative to the percentage of dry mass. To assess changes in the tissue biomass composition and their impact on the holobiont (host + symbiont) δ^13^C and δ^15^N values, an isotope mass balance model was applied following the approach outlined by Hayes (2001). The isotopic composition of the holobiont (δ^13^C_holobiont_ or δ^15^N_holobiont_) was calculated using Equation (2).

(2) $$\delta^{13}C_{\text{holobiont}}=m_{h}\times \delta^{13}C_{h}+m_{s}\times \delta^{13}C_{s}$$

where *m* represents the estimated proportion of host (*m*_h_) and symbiont (*m*_s_) tissues within the ash‐free dry weight (AFDW) of holobiont biomass, and δ^13^C, as previously defined, corresponds to the isotopic values of these tissues (Wall et al., 2019). Given that Symbiodiniaceae typically constitute 3%–10% of coral biomass (Porter et al., 1989), and considering that environmental factors may influence this percentage in ways that are not yet fully understood (Thornhill et al., 2011), a conservative estimate of 5% of the total biomass was employed in the calculations (Wall et al., 2019).

### 2.4 Isotope analysis of nutrient sources

Potential sources of heterotrophic carbon and nitrogen in corals were sampled at depths of 3 and 6 m depth at each site during two separate cruises. All POM samples were taken in triplicate. Considering that DOM samples are instrumented for multiple sampling tests and then averaged as much as the testing error allows, we thus collect approximately 20% of the replicates as a quality control for field sampling. δ^13^C_DOM_, δ^15^N_DOM_, δ^13^C_POM_, and δ^15^N_POM_ were analyzed following established protocols (e.g., Yamamoto et al., 2019; Zhang et al., 2020). Briefly, seawater samples were filtered for DOM and POM using pre‐combusted Whatman GF/F filters (0.7 μm pore size, 450°C for 5 h) (Kaldy, 2012). The DOM samples were subsampled, transferred into 40 mL acid‐washed and combusted brown glass vials (450°C for 5 h), acidified with phosphoric acid, and stored at −20°C for subsequent stable isotope analysis. The filters retaining POM were freeze‐dried at −80°C in an Ultra‐low Freeze Dryer (Biosafer‐18A, Jiangsu, China) and preserved for stable isotope analysis of the POM.

δ^13^C_DOM_ and δ^15^N_DOM_ were measured using a total organic carbon analyzer‐stable isotope mass spectrometer coupled with an EA‐IRMS (Elementar Vario PYRO cube‐IsoPrime100 Isotope Ratio Mass Spectrometer, Germany) at the TIO. The long‐term precision of the instrument is approximately ±0.2‰ for C and ±0.3‰ for N. DOC was quantified using a total organic carbon analyzer (TOC‐L CPH, Shimadzu, Kyoto, Japan) at the TMBRS. δ^13^C_POM_ and δ^15^N_POM_ were measured with an elemental analyzer combined with an isotope ratio mass spectrometer (EA‐IRMS; Inegra2, Sercon Limited, Crewe, UK) at the TIO. Carbon and nitrogen concentrations were calculated based on the GF/F filter area ratio, and subsequently, the carbon and nitrogen concentrations of the POM were determined according to the volume of the filtered seawater (Yamamoto et al., 2019). The long‐term precision for the instrument used in this analysis is about ±0.2‰ for C and ±0.25‰ for N.

Due to logistical constraints, samples for dissolved inorganic carbon (DIC) and dissolved inorganic nitrogen (DIN) were not collected (Table 1). Instead, we utilized previously reported data to estimate average δ^13^C‐DIC values for the wet and dry seasons. These values were obtained from the weekly δ^13^C‐DIC measurements conducted by Deng et al. (2013) on the Luhuitou fringing reef from January to December 2011. Additionally, the δ^15^N of DIN for the wet and dry seasons were obtained from data reported by Zhang et al. (2020) and Yang et al. (2017), respectively, for surface seawater in the South China Sea.

**TABLE 1:** Summary of mean δ^13^C and δ^15^N values (mean ± SD) and fractionation or trophic discrimination factors (TDF) for each source in the Luhuitou fringing reef during the wet and dry seasons.

	DIM		DOM		POM	
	δ^13^C (‰)	δ^15^N (‰)	δ^13^C (‰)	δ^15^N (‰)	δ^13^C (‰)	δ^15^N (‰)
The measured or estimated value						
Wet season	−2.02 ± 0.50^a^	4.60 ± 0.20^b^	−24.54 ± 0.41^c^	−1.59 ± 0.42^c^	−21.28 ± 0.87^c^	5.28 ± 1.44^c^
Dry season	−1.67 ± 0.18^a^	4.80 ± 0.30^b^	−25.16 ± 0.47^c^	−0.27 ± 0.92^c^	−23.88 ± 1.19^c^	−2.00 ± 3.55^c^
TDF	−12.10 ± 3.00^d^	0.00 ± 0.00^d^	1.00 ± 1.00^e^	2.60 ± 2.10^f^	1.00 ± 1.00^e^	2.60 ± 2.10^f^

^a^ δ^13^C of DIC was calculated from the values reported by Deng et al. (2013).

^b^ δ^15^N of DIN for the wet and dry seasons were obtained from the values reported by R. Zhang et al. (2020) and Yang et al. (2017), respectively.

^c^ This study.

^d^ Estimated fractionation values of δ^13^C and δ^15^N for DIM from Price et al. (2021) and references therein.

^e^ Estimated fractionation values of δ^13^C for DOM and POM at different trophic levels (Newsome et al., 2010).

^f^ Estimated fractionation δ^15^N values for DOM and POM at different trophic levels (Owens, 1988).

### 2.5 Bayesian mixing models

In the Bayesian mixing models applied in this study, we adopted the DIM fractionation values from Price et al. (2021) (Table 1). Specifically, the δ^13^C‐DIC was set at 12.1‰ ± 3.0‰ and the δ^15^N‐DIN was set at 0.00‰. For mean trophic discrimination factor (TDF) for δ^13^C across heterotrophic sources (i.e., POM and DOM), a value of 1.0 ± 1.0‰ was used, drawing on measurements from marine predators due to the lack of published TDF estimates specific to coral heterotrophic sources (Newsome et al., 2010). The selection of TDFs for δ^15^N in DOM and POM is crucial for the accuracy of our MixSIAR model. In this study, the application of the MixSIAR model to estimate the contributions of heterotrophic sources to coral nutrition followed the approach of Price et al. (2021). In their study, a TDF of 3.4‰ ± 1.0‰ for δ^15^N of the same heterotrophic sources (POM and Zooplankton) was chosen based on extensive prior measurements of nitrogen isotopes enrichment across trophic levels (Newsome et al., 2010; Post, 2002).

Given the uncertainty surrounding the TDF value for symbiotic corals utilizing heterotrophic sources, Price et al. (2021) reduced the TDF values of heterotrophic sources to 0.0‰ ± 0.0‰ and ran the model again to account for the fact that carbon and nitrogen cycling between the host and symbionts may eliminate or reduce any trophic enrichment of these isotopes. When the TDF value was set to zero, the model results showed that the mean estimated contribution of heterotrophy increased by only 2.5% ± 4.2%. Despite this adjustment, DIM still accounted for approximately two‐thirds of the estimated contribution of coral tissue on average.

In Price et al. (2021), the model results using a TDF of 3.4‰ ± 1.0‰ for nitrogen served as the primary basis for analysis and discussion. Price et al. (2021) explicitly endorsed the model outcomes associated with this nitrogen TDF value. Notably, the TDF values for δ^15^N between trophic levels referenced in Price et al. (2021) were derived from Post (2002). However, Post's (2002) study obtained mean TDF values for δ^15^N between trophic levels were obtained from samples collected in 25 north temperate lakes. In contrast, Owens (1988) reported a mean TDF value of 2.60‰ ± 2.10‰ for δ^15^N between trophic levels in marine ecosystems. This comparison highlights the variation in TDF values for δ^15^N between different trophic levels across marine ecosystems and lake ecosystems.

Given the uncertainty surrounding the TDF values for corals utilizing heterotrophic sources and their growth within the marine environments, this study chose to use TDF values for δ^15^N in DOM and POM, and we chose to run the model with a value that is lower than the value reported by Price et al. (2021), that is, 2.60‰ ± 2.10‰ as reported by Owens (1988). The TDF values for the DIM (i.e., DIC + DIN) source values remained unchanged in the Bayesian mixing models (Price et al., 2021).

### 2.6 Photosynthesis–irradiance (P–I) curve of *G. fascicularis*

The Photosynthesis–irradiance (P–I) curves of *G. fascicularis* were determined using a multichannel fiber‐optic oxygen meter (OXY‐4 mini) from PreSens (Regensburg, Germany). On June 13, 2018, 10 fragments (each approximately 1.5 cm^2^ each) of healthy *G. fascicularis* were collected from a depth of approximately 3 m near site C3 at the Tropical Marine Biological Research Station in Hainan, Chinese Academy of Sciences (Figure 1). Two 400 W metal halide lamps (Philips, HPIT) were employed as PAR sources, with the intensity of PAR measured using a spherical quantum sensor (Li‐Cor LI‐193, Lincoln, NE, USA). A PAR intensity gradient was established by controlling the distance between the coral fragments and the metal halide lamp, resulting in the following intensities: 0, 15, 30, 60, 100, 200, 400, 600, and 1000 μmol photons m^−2^ s^−1^, respectively. The net photosynthetic rate (*P*_net_) of each coral fragment at different intensities of PAR was measured using a calibrated OXY‐4 mini with the water temperature maintained at 27°C. The P–I curve was generated by fitting the data to the following Equation (3):

(3) $$P_{\mathrm{net}}=P_{\max}^{g}\tanh\left(I/\mathrm{Ik}\right)+R$$

where *P*_net_ is the net photosynthetic rate in nmol O_2_ cm^−2^ min^−1^, $P_{\max}^{g}$ is the maximum rate of gross photosynthesis in nmol O_2_ cm^−2^ min^−1^, *I* is the light intensity of the low‐light threshold in μmol photons m^−2^ s^−1^, *Ik* is the saturation light intensity, that is, the minimum light intensity at $P_{\max}^{g}$ in μmol photons m^−2^ s^−1^, and *R* is the dark respiration rate in nmol O_2_ cm^−2^ min^−1^.

### 2.7 Statistical analyses

Sampling station maps were generated using Golden Software Surfer V13.0 (Golden, CO, USA). The Bayesian stable isotope mixing models were conducted with the “MixSIAR” package in R (version 4.2.1 for Windows; https://CRAN.R‐project.org/) package “MixSIAR” (Stock et al., 2018) to estimate the contribution of various nutrient sources to corals. Pearson's correlation analysis between coral isotopic compositions and environmental variables, including light, was visualized using Origin 2022 (OriginLab Corporation, Wellesley, MA, USA), with statistical significance set at *p* ≤ .05. To evaluate significant differences in the mean carbon and nitrogen isotope values of coral hosts and symbionts across different seasons and depths, a one‐way ANOVA (*p* < .05) was conducted using SPSS Statistics 27 (IBM SPSS, Chicago, IL, USA). Photosynthesis–irradiance (P–I) curve fitting was plotted using GraphPad Prism 9.0 (GraphPad Software, San Diego, CA, USA), and the final figures were assembled using Adobe Illustrator 2021 software (Adobe, San Jose, CA, USA).

## 3 RESULTS

### 3.1 δ^13^C, δ^15^N, and C:N ratios of coral hosts and symbionts

**FIGURE 2:**
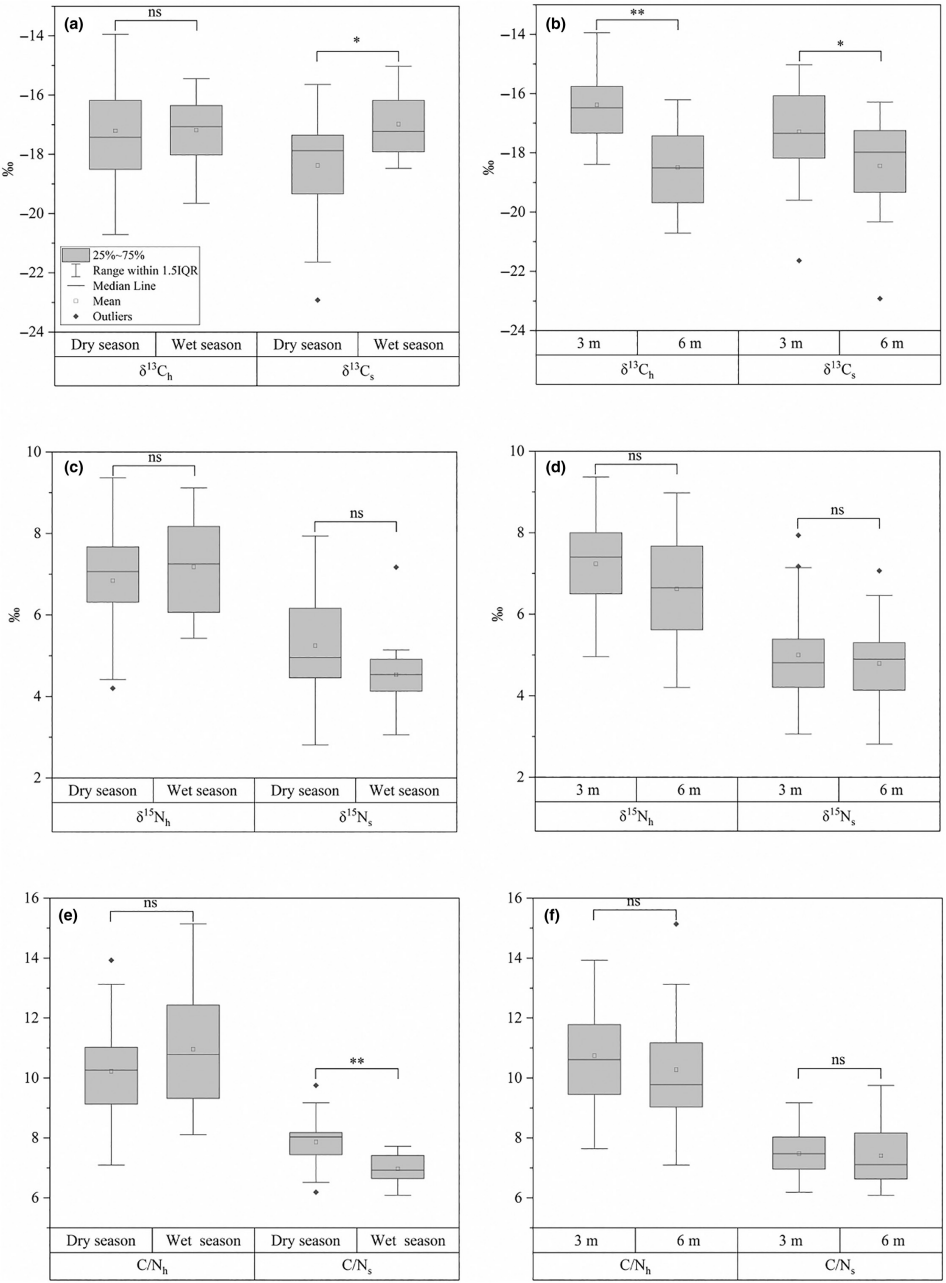
Box plots summarizing the averaged overall data for δ^13^C, δ^15^N, and C/N of coral hosts and symbionts across different seasons (a, c, e) and depths (b, d, f). Each box plot includes data from *n* = 18 samples for the wet season and *n* = 21 samples for the wet and dry seasons, as well as *n* = 24 samples at 3 m depth and *n* = 15 samples at 6 m depth. The plots display medians (central horizontal lines), interquartile ranges (boxes), 95% confidence intervals (whiskers), and outliers (solid diamonds). The asterisks indicate significant differences (*p < .05, **p < .01), while “ns” indicates no significant differences.

The mean δ^13^C values of the coral hosts (δ^13^C_h_) did not show significant seasonal variation (Figure 2a). However, individuals sampled at a depth of 6 m displayed significantly more negative δ^13^C_h_ values compared to those at 3 m (*p* < .01) (Figure 2b). Additionally, the mean δ^13^C values of the symbionts (δ^13^C_s_) were significantly more negative during the dry season (*p* < .05) and were also significantly lower at 6 m compared to 3 m (*p* < .05) (Figure 2a,b). Furthermore, at 3 m depth, the mean value of δ^13^C_h_ was significantly more positive than that of δ^13^C_s_ for samples taken at 3 m depth (*p* < .05) (Figure S1).

No significant differences were observed in the mean δ^15^N values of the coral hosts (δ^15^N_h_) or symbionts (δ^15^N_s_) across either season or depth (Figure 2c,d). However, when comparing the coral hosts to their symbionts, δ^15^N_h_ values were consistently more positive than δ^15^N_s_ values, regardless of season or depth (*p* < .01) (Figure S1).

The mean C/N ratios of coral hosts (C/N_h_) did not show significant differences across seasons or depths. In contrast, while the mean C/N ratios of symbionts (C/N_s_) were significantly higher in the wet season compared to in the dry season (*p* < .001) (Figure 2e,f). Similar to the δ^15^N values, the C/N_h_ ratios were consistently higher than the C/N_s_ ratios, with this relationship remaining stable across both season and depth (Figure S1).

### 3.2 δ^13^C, δ^15^N, and C/N for DOM and POM

The mean δ^13^C_DOM_ value was significantly more positive in the wet season compared to in the dry season (*p* < .01); there was no significant variation by depth (Table 2). The mean δ^15^N_DOM_ value also differed significantly between the wet and dry seasons (*p* < .01), with higher values observed in the dry season, and no significant difference was found with respect to depth. Notably, although δ^13^C_POM_, δ^15^N_POM_, and C/N_POM_ values showed no significant differences between 3 and 6 m depths, the mean δ^13^C_POM_ and δ^15^N_POM_ values were significantly higher in the wet season than in the dry season (*p* < .01), and the mean C/N_POM_ values were lower in the wet season (*p* < .01) (Table 2).

**TABLE 2:** Mean (±SD) and one‐way ANOVA results for δ^13^C, δ^15^N, and C:N of DOM and POM.

	δ^13^C (‰)		δ^15^N (‰)		C/N (mole mole^−1^)	
	Mean ± SD	*n*	Mean ± SD	*n*	Mean ± SD	*n*
DOM						
Total data	−24.90 ± 0.53	16	−0.88 ± 1.06	16	/	/
Wet season	−24.44 ± 0.24	8	−1.66 ± 0.43	8	/	/
Dry season	−25.35 ± 0.25	8	−0.11 ± 0.92	8	/	/
Surface water	−24.92 ± 0.57	8	−0.54 ± 1.23	8	/	/
Bottom water	−24.87 ± 0.52	8	−1.23 ± 0.79	8	/	/
POM						
Total data	−22.58 ± 1.67	48	1.64 ± 4.55	48	5.51 ± 1.80	48
Wet season	−21.28 ± 0.87	24	5.28 ± 1.44	24	4.27 ± 1.40	24
Dry season	−23.88 ± 1.19	24	−2.00 ± 3.55	24	6.75 ± 1.22	24
Surface water	−22.45 ± 1.74	24	1.63 ± 5.06	24	5.52 ± 2.04	24
Bottom water	−22.72 ± 1.63	24	1.66 ± 4.09	24	5.51 ± 1.57	24
Significance test						
DOM						
Seasons	***p* < .001** ^**^		***p* = .001** ^**^		/	
Depths	*p* = .861		*p* = .205		/	
POM						
Seasons	***p* < .001** ^**^		***p* < .001** ^**^		***p* < .001** ^**^	
Depths	*p* = .510		*p* = .978		*p* = .988	

*Note*: Significant results (*p* < .05 and *p* < .01) are shown in bold with an asterisk (*) and two asterisks (**), respectively.

Abbreviations: DOM, dissolved organic matter; POM, particulate organic matter.

### 3.3 Contribution of DIM, DOM, and POM to whole coral tissues

**FIGURE 3:**
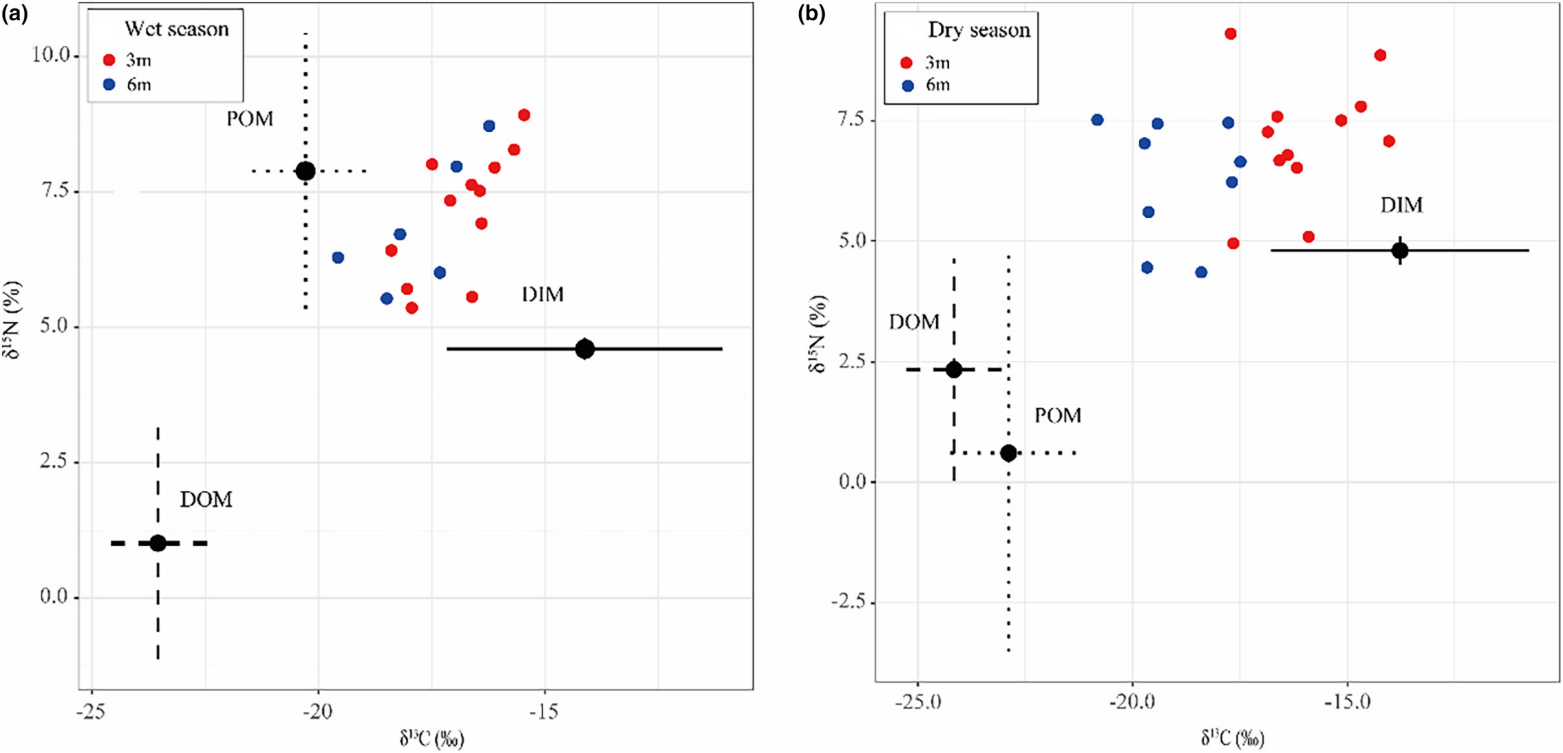
Isotope biplot of the coral *G. fascicularis* at each depth on the Luhuitou fringing reef during the wet season (a) and dry season (b). Each source (DIM, DOM, and POM) is plotted with fractionation and trophic discrimination factors considered (Table 1). DIM, dissolved inorganic matter; DOM, dissolved organic matter; POM, particulate organic matter.

**FIGURE 4:**
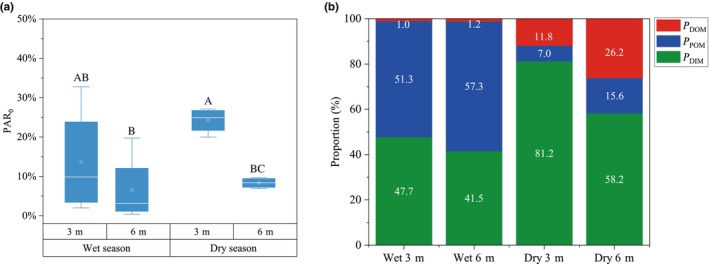
Variations in light intensity across seasons and depths along with the corresponding nutrient status of the corals. (a) Box plots of the average summary statistics of all data for light intensity (as a percentage relative to surface photosynthetically active radiation, %PAR_0_) for different seasons and depths. Each box plot represents data from *n* = 4 samples. The box plots include medians (central horizontal lines), interquartile ranges (boxes), and 95% confidence intervals (whiskers). Significant differences are indicated by different letters above the bars (*p* < .05, one‐way ANOVA followed by LSD post hoc test); (b) stacked bar chart shows the percentage contribution of dissolved organic matter (DOM), particulate organic matter (POM), and dissolved inorganic matter (DIM) to coral nutrition.

**FIGURE 5:**
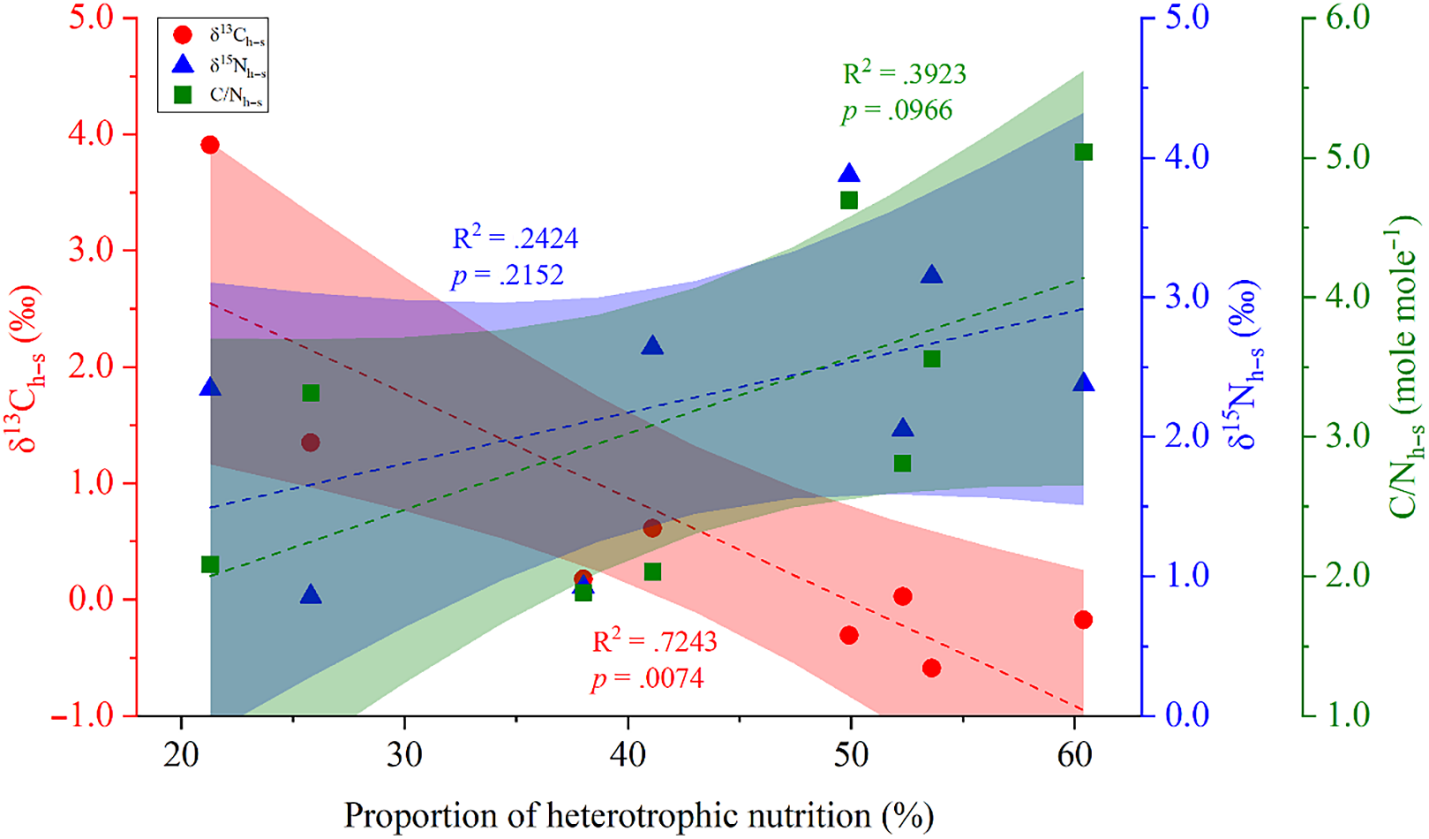
Correlations between the proportion of heterotrophic nutrition and δ^13^C_h–s_ (red solid circles), δ^15^N_h–s_ (blue solid triangles), and C/N_h–s_ (green solid squares), respectively. Each data point represents the mean value of samples collected at each depth across the study. The correlations were fitted using a linear model with a 95% confidence interval (*n* = 8), and a significant level of *p* < .05 was applied to determine statistical significance.

The relative contribution of each nutrient source to the coral tissue was estimated using MixSIAR model (Table 1 and Figure 3). Significant differences were observed in the contribution of each nutrient source to corals under varying levels of PAR. The highest estimated mean contribution from autotrophy (*P*_DIM_) to the corals was 81.2% at 24.23% PAR_0_, while the lowest was 41.5% at 6.60% PAR_0_, respectively (Figure 4, Table S1). During the wet season, the contribution of POM to heterotrophic nutrition (*P*_POM_) ranged from 51.3% to 57.3%, significantly higher than that of DOM, which ranged from 1.0% to 1.2%. In contrast, in the dry season, the contribution of DOM to heterotrophic nutrition (*P*_DOM_) was significantly higher, ranging from 11.8% to 26.2%, compared to POM, which ranged from 7.0% to15.6% (Figure 4, Table S1). In addition, the mean values of δ^13^C_h–s_ values were 0.91‰ at 3 m depth and −0.05‰ at 6 m depth, while the mean values of δ^15^N_h–s_ values were 2.24‰ and 1.83‰ at 3 and 6 m depths, respectively (Table S2). Notably, the heterotrophic contribution was significantly negatively correlated with δ^13^C_h–s_, whereas the correlations with δ^15^N_h–s_ and C/N_h–s_ were not significant (Figure 5).

### 3.4 Correlations between %PAR_0_ and δ^13^C, δ^15^N, and C/N of the coral

**FIGURE 6:**
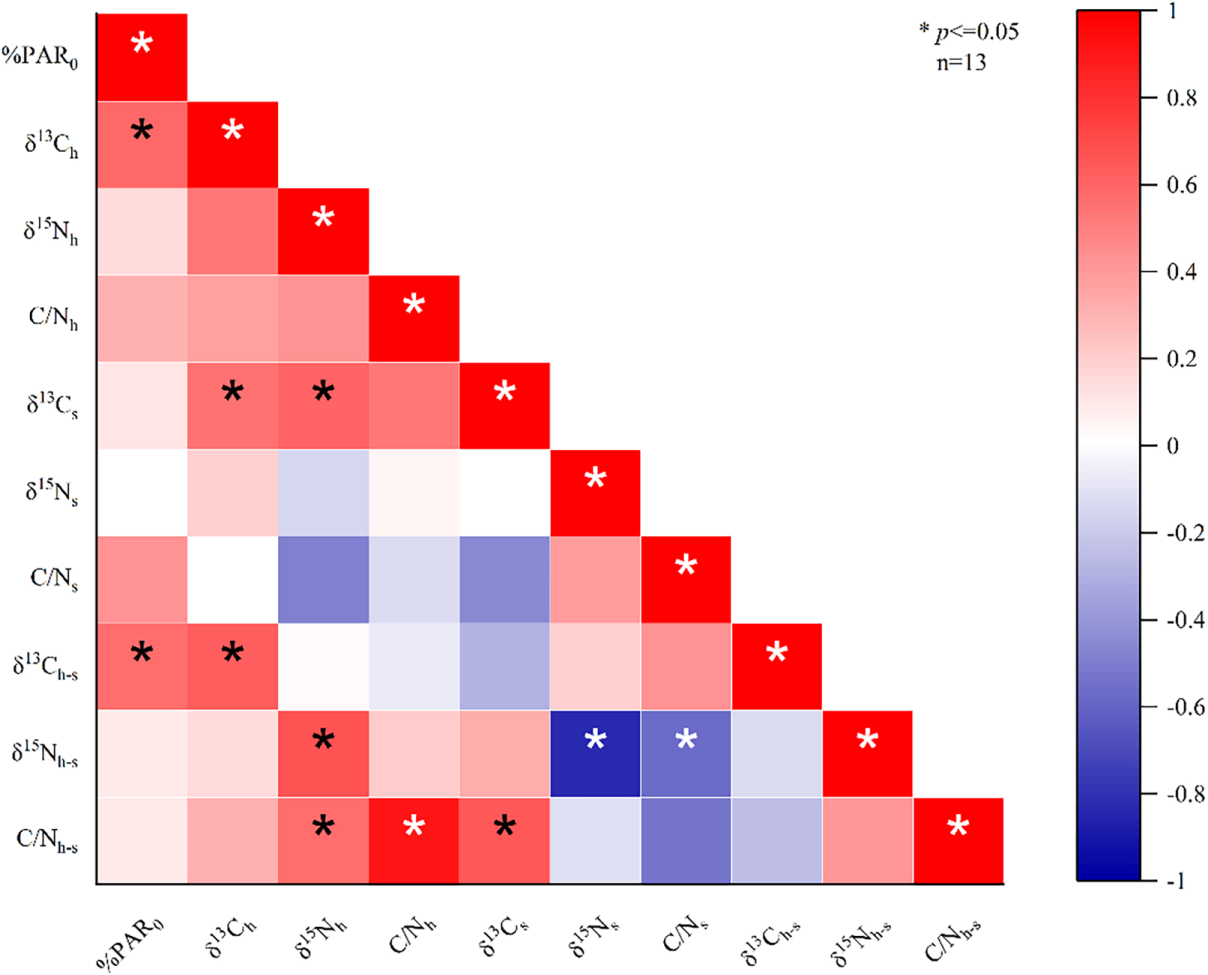
Correlations between %PAR_0_ and δ^13^C, δ^15^N, and C/N ratios of the coral *G. fascicularis* (host and symbiont). In this matrix, red squares indicate positive correlations, while blue squares denote negative correlations. Asterisks represent significant correlations (*p* ≤ .05). %PAR_0_: Percentage of surface photosynthetically active radiation.

Importantly, the %PAR_0_ values obtained at 3 and 6 m depths were found to have significant positive correlations with δ^13^C_h_ and δ^13^C_h–s_. However, no significant correlations were observed for δ^15^N_h_, C/N_h_, δ^13^C_s_, δ^15^N_s_, C/N_s_, δ^15^N_h–s_, or C/N_h–s_ (*p* ≤ .05) (Figure 6). Additionally, δ^13^C_h_ was significantly positively correlated with δ^13^C_s_, δ^15^N_h‐s_, and C/N_h–s_ (*p* ≤ .05). A significant positive correlation was also observed between C/N_h_ and C/N_h–s_ (*p* ≤ .05), while no significant correlation was observed between C/N_s_ and C/N_h–s_. Notably, δ^15^N_h–s_ was significantly negatively correlated with both δ^15^N_s_ and C/N_s_ (*p* ≤ .05) (Figure 6).

### 3.5 Correlation of heterotrophic sources with their contribution to coral nutrition and with other environmental factors

**FIGURE 7:**
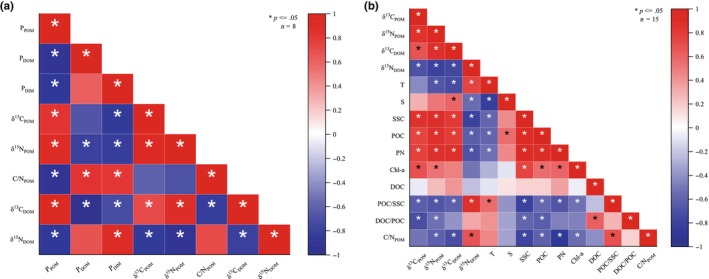
Correlation of heterotrophic sources with their contribution to coral nutrition (a) and with other environmental factors (b). In this matrix, red squares indicate positive correlations, while blue squares denote negative correlations. Asterisks represent significant correlations (*p* ≤ .05); *P*_POM_ and *P*_DOM_ indicate the proportion of POM and DOM in coral nutrition, respectively. Chl‐a, Chlorophyll a; DOC, dissolved organic carbon; DOM, dissolved organic matter; PN, particulate nitrogen; POC, particulate organic carbon; POM, particulate organic matter; S, salinity; SSC, suspended solids concentration; T, Temperature.

*P*_POM_ exhibited a significant positive correlation with both δ^13^C_POM_ and δ^15^N_POM_, and a significant negative correlation with C/N_POM_ (*p* ≤ .05) (Figure 7a). Conversely, *P*_DOM_ demonstrated a significant negative correlation with δ^13^C_DOM_ (*p* ≤ .05), whereas the correlation with δ^15^N_DOM_ was not significant.

Although δ^13^C_POM_ exhibited significant positive correlations with SSC, POC, PN, and Chl‐a, it showed a significant negative correlation with the ratios of POC/SSC and DOC/POC (*p* ≤ .05) (Figure 7b). In contrast, the value of δ^15^N_POM_ showed significant positive correlations with SSC, POC, PN, and Chl‐a and significant negative correlations with T, POC/SSC, and DOC/POC (*p* ≤ .05).

The δ^13^C_DOM_ showed significant positive correlations with S, SSC, POC, and PN while showing a significant negative correlation with both T and POC/SSC ratio (*p* ≤ .05) (Figure 7b). Conversely, δ^15^N_DOM_ demonstrated significant positive correlations with T and POC/SSC (*p* ≤ .05) but showed significant negative correlations with S, SSC, POC, and PN (*p* ≤ .05).

### 3.6 Correlation between light intensity and coral cover

The shipboard survey results showed that the mean PAR at 3 m depth (18.93 ± 10.94%PAR_0_) was significantly higher compared to 6 m depth (7.47 ± 5.97%PAR_0_) (*p* < .05) (Table 3). Additionally, a significant difference in *G. fascicularis* cover was observed between these depths (*p* < .05), with a mean cover of 5.39 ± 4.16%. *G. fascicularis* cover constituted 14.89 ± 9.49% of the total coral cover and was significantly higher at 3 m compared to at 6 m (*p* < .001) (Table 3).

**TABLE 3:** Mean (±SD) and one‐way ANOVA results for %PAR0, *G. fascicularis* cover and *G. fascicularis*/total coral cover.

	%PAR_0_		*G. fascicularis* cover (%)		*G. fascicularis*/total coral cover (%)	
	Mean ± SD	*n*	Mean ± SD	*n*	Mean ± SD	*n*
Total data	13.20 ± 10.37	16	5.39 ± 4.16	6	14.89 ± 9.49	6
3 m	18.93 ± 10.94	8	8.83 ± 2.68	3	23.47 ± 0.47	3
6 m	7.47 ± 5.97	8	1.95 ± 0.75	3	6.30 ± 1.95	3
Significance test	***p* = .021** ^**^		***p* = .013** ^**^		***p* < .001** ^**^	

*Note*: Significant results (*p* < .05 and *p* < .01) are shown in bold with an asterisk (*) and two asterisks (**), respectively.

Abbreviation: %PAR_0_, percentage of surface photosynthetically active radiation.

**FIGURE 8:**
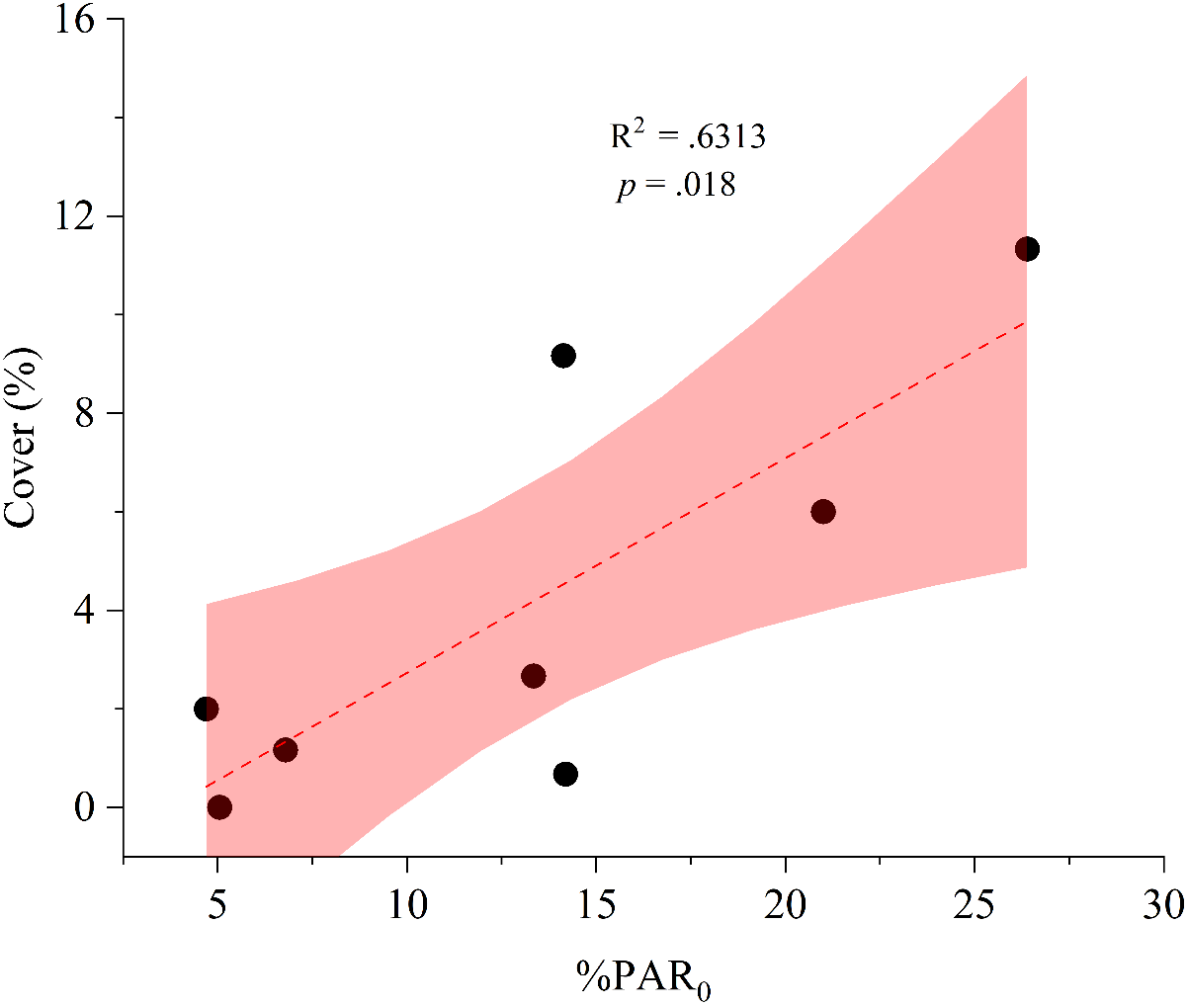
Correlation between %PAR_0_ and the cover of coral *G. fascicularis*. Each data point represents the light intensity corresponding to the *G. fascicularis* cover at various depths and sites. The correlation was fitted with a linear model with 95% confidence interval (*n* = 8), and significance was determined at *p* < .05. %PAR_0_, Percentage of surface photosynthetically active radiation.

Notably, %PAR_0_ exhibited a significantly positive correlation with the cover of *G. fascicularis* cover (*p* < .05) (Figure 8). The correlation analysis indicated that the low‐light threshold for *G. fascicularis* growth was approximately 3.73%PAR_0_. Additionally, the maximum depth (MD) of *G. fascicularis* on the Luhuitou fringing reef ranged from 5.98 to 9.67 m, with a mean value of 7.31 m (Figure S2).

### 3.7 Photosynthesis‐irradiance curves of *G. fascicularis* on the Luhuitou fringing reef

**FIGURE 9:**
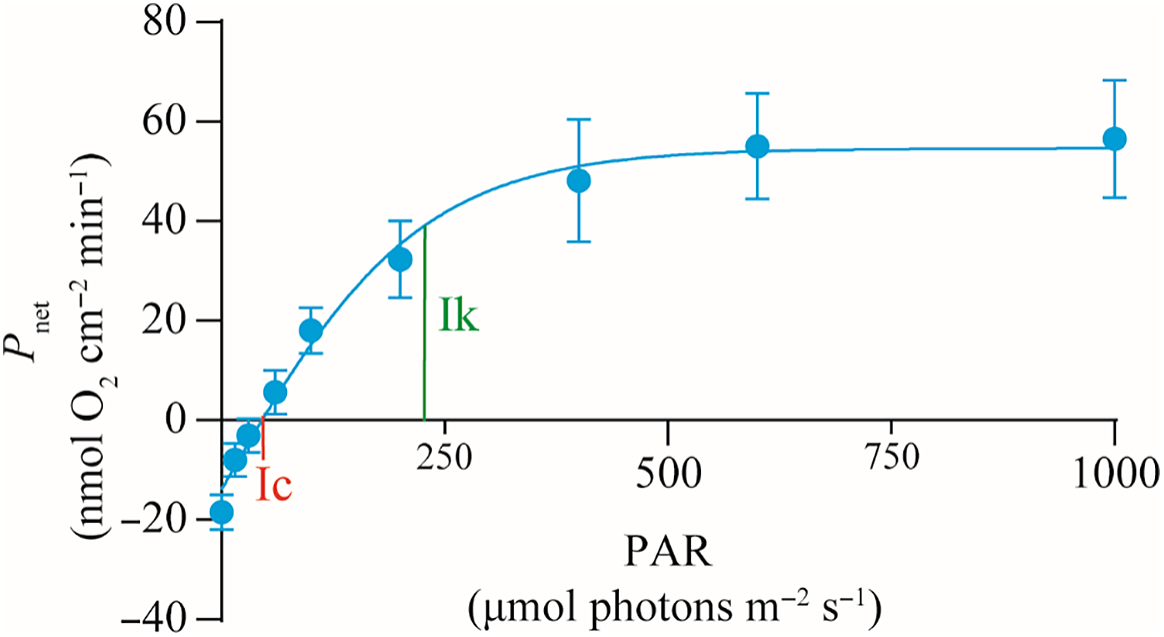
Photosynthesis–irradiance (P–I) curve for the coral *G. fascicularis* at a depth of 3 m on the Luhuitou fringing reef. P–I curve fitting was performed using the GraphPad Prism 9.0. Error bars indicate the standard error of the mean for the fitted values. Ic, light compensation point; Ik, light saturation point; PAR, photosynthetically active radiation; *P*_net_, net photosynthetic rate.

Based on the photosynthesis–irradiance (P–I) curve, the initial light intensities required to reach the light compensation point (Ic) and the light saturation point (Ik) of the coral *G. fascicularis* were 45.61 and 221.10 μmol photons m^−2^ s^−1^, respectively (Figure 9).

## 4 DISCUSSION

Low‐light environments are driving vertical reef compression and favoring depth generalists within coral communities lying on inshore turbid reefs, highlighting an emerging local threat that is affecting fringing reefs on a global scale (Morgan et al., 2020; Zweifler et al., 2021). This issue is exacerbated by the increase in suspended solids in tropical coastal waters due to urban, agricultural, and industrial runoff, as well as deforestation, resuspension, and dredging activities (Magris et al., 2019; Zweifler et al., 2021). Our study provides direct field evidence that the reef coral *G. fascicularis* exhibits heterotrophic plasticity, selectively utilizing heterotrophic sources based on their bioavailability, which in turn enhances its heterotrophic contribution, bolstering its resistance to low‐light conditions. These findings suggest that reef‐building corals with such heterotrophic plasticity may have a greater potential to persist or even dominate in environments affected by coastal darkening.

### 4.1 Heterotrophic compensation of coral nutrition under reduced light availability

Previous studies have demonstrated that variations in δ^13^C values of coral tissues can serve as indicators of whether their nutrition is predominantly autotrophic or heterotrophic (Heikoop et al., 2000; Nahon et al., 2013). In our study, the δ^13^C values of the coral host (δ^13^C_h_) and its symbionts (δ^13^C_s_) ranged from −20.71‰ to −13.9 5‰ and −22.92‰ to −15.03‰, respectively. According to previous research, the rate of photosynthesis in corals is influenced by light intensity, with higher photosynthetic rates leading to heavier carbon isotope ratios (i.e., ^13^C‐enriched) in coral tissues (Muscatine et al., 1989; Xu et al., 2020). This implies that some of the changes in δ^13^C values in coral tissues may be attributed to variations in photosynthesis rates rather than shifts in diet. Theoretically, δ^13^C_h_ and δ^13^C_s_ values should be similar, or δ^13^C_h_ values might be slightly higher than δ^13^C_s_, indicating a minimal or negligible contribution of heterotrophy to coral carbon fixation (Muscatine et al., 1989). However, when the heterotrophic contribution to coral nutrition increases, δ^13^C_h_ values decrease and approach those of ^13^C‐depleted heterotrophic sources (e.g., zooplankton and POM), which typically range from −14‰ to −25‰ or even lower (Xu et al., 2020), resulting in significantly negative δ^13^C_h–s_ (δ^13^C differences between δ^13^C_h_ and δ^13^C_s_). Our findings reveal that while the correlation between %PAR_0_ and δ^13^C_s_ was not significant, there was a significant positive correlation between δ^13^C_h_ and δ^13^C_s_. Importantly, the range of values for δ^13^C_h_ and δ^13^C_s_ values observed in our study is not only significantly more negative than the characteristic δ^13^C values of autotrophic‐dominated corals (typically −14.0‰ to −10.0‰) (Table S2) but also closely aligns with the δ^13^C values of ^13^C‐depleted heterotrophic sources with δ^13^C < −16‰ (i.e., POM and DOM) (Heikoop et al., 2000; Muscatine et al., 1989). Furthermore, we found that the mean value of δ^13^C_h–s_ value at 6 m was −0.05‰. These results indicate that *G. fascicularis* exhibits significant trophic plasticity, with the observed decrease in δ^13^C_s_ likely driven by increased reliance on heterotrophic nutrition.

The results of our MixSIAR model revealed that the heterotrophic contribution to coral nutrition was at its lowest (18.8%) when underwater light intensity was highest (24.23% PAR_0_) and reached its peak (58.5%) under the lowest light conditions (6.60% P AR_0_). This maximum heterotrophic contribution under low‐light conditions exceeds the typical range within healthy corals to meet 5%–50% of their metabolic demand through heterotrophy (Grottoli et al., 2006; Price et al., 2021). Considering that certain coral species are known to increase their reliance on heterotrophic carbon under environmental stress (Baumann et al., 2014; Hughes & Grottoli, 2013), our findings suggest that the increased heterotrophic contribution observed in *G. fascicularis* under low‐light conditions may be a response to light stress. However, our results contrast with those of Martinez et al. (2020), who reported that the proportions of heterotrophic and autotrophic contributions to the nutrition of the coral *Stylophora pistillata* remained consistent across well‐lighted environment of the shallow water and the lower‐lighted mesophotic zone. Notably, their study found that heterotrophy consistently accounted for approximately 35% of the coral's nutritional intake, regardless of depth and light conditions. In contrast, our study demonstrates that changes in PAR significantly influenced the heterotrophic contribution in corals, which increased with decreasing PAR, indicating that heterotrophy was not stable but rather characterized by low PAR and high heterotrophic contribution. This discrepancy highlights a fundamental difference in the nutrient acquisition strategies between *S. pistillata* and *G. fascicularis* under low light conditions. A key factor may be that as PAR decreased, *S. pistillata* maintained a higher autotrophic contribution through photoacclimatization, allowing it to sustain the balance between autotrophic and heterotrophic contributions as PAR decreases (Carpenter et al., 2022; Mass et al., 2007). In contrast, *G. fascicularis* compensate for reduced autotrophic input by increasing its reliance on heterotrophy to meet its metabolic nutrient requirements.

Furthermore, the heterotrophic contribution to nutrition of *G. fascicularis* was found to be significantly negatively correlated with δ^13^C_h–s_ values. This result aligns with the findings of Muscatine et al. (1989) and Xu et al. (2020), who reported that lower δ^13^C_h–s_ values indicate a greater reliance on heterotrophy for carbon fixation compared to photosynthesis. However, no significant correlation was observed between the heterotrophic contribution and δ^15^N_h–s_ (the difference between δ^15^N_h_ and δ^15^N_s_). This result contrasts with the findings of Conti‐Jerpe et al. (2020) and Price et al. (2021), who suggested that δ^15^N_h–s_ is a more reliable indicator of heterotrophic contribution to coral nutrition than δ^13^C_h–s_. In our study, δ^13^C_h–s_ was significantly positively correlated with δ^13^C_h_ and showed no significant correlation with δ^13^C_s_, while δ^15^N_h–s_ was significantly positively correlated with δ^15^N_h_ and significantly negatively correlated with both δ^15^N_s_ and C/N_s_. These results suggest that the inconsistent variations in the δ^13^C_h–s_ and δ^15^N_h–s_ values, which reflect the heterotrophic contribution to coral *G. fascicularis*, may be primarily related to isotopic fractionation effects arising from different turnover rates of carbon and nitrogen acquired through heterotrophy (Tanaka et al., 2018). Consequently, our results imply that δ^13^C_h–s_ values effectively characterize the heterotrophic contribution in *G. fascicularis* when the ratio of autotrophic to heterotrophic nutrients fluctuates due to changes in PAR.

### 4.2 Effect of heterotrophic resource availability on its contribution

In this study, the average contribution of POM and DOM to the nutrition of *G. fascicularis* nutrition ranged from 7.0% to 57.3% and 1% to 26.2%, respectively. These findings align with previous research, which underscores the importance of both POM and DOM as critical heterotrophic sources for corals (Houlbreque & Ferrier‐Pages, 2009). Notably, the contribution of POM to *G. fascicularis* nutrition exhibited a significant positive correlation with δ^13^C_POM_ and δ^15^N_POM_, while being negatively correlated with C/N_POM_. Conversely, the contribution of DOM to *G. fascicularis* nutrition was significantly negatively correlated with the δ^13^C_DOM_ but showed no significant correlation with δ^15^N_DOM_. These results highlight the presence of significant seasonal variations in the respective contributions of POM and DOM to *G. fascicularis* nutrition and suggest that these differences may be influenced by the seasonal bioavailability of POM and DOM.

The lower contribution of POM to *G. fascicularis* nutrition in the dry season aligns with the observation that the proportion of terrestrial‐derived POM was three times higher in this season compared to the wet season (Luo et al., 2022). This finding corroborates recent studies indicating that corals struggle to digest and utilize terrestrial‐derived POM, such as microplastics (Reichert et al., 2022; Savinelli et al., 2020). Additionally, in comparison to the wet season, the δ^13^C_DOM_ values were significantly more negative, and the δ^15^N_DOM_ values were significantly more positive in the dry season. Given that urban sewage discharge can lead to a decrease in δ^13^C_DOM_ and an increase in δ^15^N_TDN_ (TDN: total dissolved nitrogen) (Delpech et al., 2021; Zhou et al., 2018), these results suggest substantial seasonal differences in the DOM sources, with a higher proportion of terrestrial‐derived DOM entering the water during the dry season. Previous research has shown that terrestrial‐derived DOM, including urea, dissolved free amino acids (DFAAs), and other small organic molecules, is highly bioavailable and can be readily absorbed and utilized by corals (Crandall & Teece, 2011; Grover et al., 2008). Therefore, the increased contribution of DOM to coral nutrition during the dry season may be attributed to the increased bioavailability of DOM. Collectively, these results demonstrate that *G. fascicularis* can selectively feed on heterotrophic sources based on their bioavailability.

### 4.3 Low‐light threshold of the *G. fascicularis* and prospects for future research

Photosynthesis serves as the primary source of energy and nutrients for shallow‐water reef corals (Iluz & Dubinsky, 2015), with the optimal light requirement for photosynthesis varying among coral species (Canto et al., 2021; Juhi et al., 2021). In our study, we observed that the reef coral *G. fascicularis* achieved its maximum total photosynthetic rate ($P_{\max}^{g}$) at a minimum PAR value of 221.1 μmol photons m^−2^ s^−1^, corresponding to the initial value of light‐saturated photosynthesis (Ik) for *this species*. Importantly, %PAR_0_ was significantly and positively correlated with *G. fascicularis* cover. Based on the significant negative correlation between *G. fascicularis* cover and %PAR_0_, the light intensity at which *G. fascicularis* cover approached zero was approximately 3.73%PAR_0_. This value is slightly higher than the Ic value of 2.05%PAR_0_ for *G. fascicularis*, indicating that there was almost no net accumulation of photosynthetic products after respiratory depletion of *G. fascicularis* at a light intensity of 3.73%PAR_0_.

In other words, the low‐light threshold required for survival of *G. fascicularis* on the Luhuitou fringing reef is approximately 3.73%PAR_0_. Based on this estimated low‐light threshold, the maximum depth at which *G. fascicularis* was observed on the Luhuitou fringing reef ranged from 5.98 and 9.67 m, which is significantly shallower than the depth of 30 m at which *G. fascicularis* was recorded on the central Maldives fore‐reef in the Indian Ocean, which extended to 30 m depth (Radice et al., 2019). These results demonstrate that while *G. fascicularis* enhances its low‐light resistance through heterotrophic compensation, it still requires a minimum level of photoautotrophic input for survival. This finding highlights the importance and urgency of developing effective measures to mitigate the low‐light conditions that threaten reef corals on inshore turbid reefs.

Furthermore, the current widespread coral bleaching driven by global warming is occurring concurrently with coastal darkening, a consequence of increased coastal development (Barlow et al., 2018; Zweifler et al., 2021). Although there is growing evidence that turbid reefs may exhibit greater resilience to global warming impacts and could serve as critical conservation hotspots (Cacciapaglia & van Woesik, 2016; Sully & van Woesik, 2020), the specific mechanisms that confer resistance to bleaching among different coral species remain poorly understood. This gap in knowledge is partly due to the difficulty in quantifying the contribution of heterotrophic sources to coral nutrition, particularly under conditions of increased light attenuation, which is especially challenging for deeper coral species (Conti‐Jerpe et al., 2020; Price et al., 2021). Recent studies using Compound‐Specific Isotope Analysis of Amino Acids (CSIA‐AA) have revealed that coral hosts can supply nutrients from heterotrophic sources to their symbionts, especially under stressful conditions (Fox et al., 2019; Goodbody‐Gringley et al., 2024; Martinez et al., 2020). CSIA‐AA offers a more precise method for identifying the diversity of trophic strategies employed by corals compared to traditional bulk stable isotope analysis (Price et al., 2021). Further studies with larger sample sizes on regional or global scales using advanced techniques like CSIA‐AA are needed to better elucidate key pathways that regulate the coupled (synergistic or antagonistic) effects of coastal darkening and global warming on coral trophic strategies. Such research will provide valuable insights into how coral communities respond to and cope with multiple stressors, facilitating the development of more effective conservation and management strategies for coral reefs in the context of ongoing climate change and coastal development.

## AUTHOR CONTRIBUTIONS

**Yong Luo:** Conceptualization (equal); investigation (equal); methodology (equal); visualization (equal); writing – original draft (equal); writing – review and editing (equal). **Xiaolei Yu:** Conceptualization (equal); investigation (equal); methodology (equal); visualization (equal); writing – original draft (equal); writing – review and editing (equal). **Lintao Huang:** Conceptualization (equal); formal analysis (equal); visualization (equal); writing – original draft (equal); writing – review and editing (equal). **Jianfeng Gan:** Conceptualization (equal); formal analysis (equal); visualization (equal); writing – original draft (equal); writing – review and editing (equal). **Xinming Lei:** Conceptualization (equal); formal analysis (equal); visualization (equal); writing – original draft (equal); writing – review and editing (equal). **Lei Jiang:** Formal analysis (equal); software (equal); writing – review and editing (equal). **Chengyue Liu:** Formal analysis (equal); software (equal); writing – review and editing (equal). **Youfang Sun:** Formal analysis (equal); software (equal); writing – review and editing (equal). **Meng Cheng:** Formal analysis (equal); investigation (equal); visualization (equal); writing – review and editing (equal). **Yuyang Zhang:** Formal analysis (equal); investigation (equal); visualization (equal); writing – review and editing (equal). **Guowei Zhou:** Methodology (equal); visualization (equal); writing – review and editing (equal). **Sheng Liu:** Methodology (equal); visualization (equal); writing – review and editing (equal). **Jiansheng Lian:** Methodology (equal); visualization (equal); writing – review and editing (equal). **Hui Huang:** Funding acquisition (lead); project administration (lead); resources (lead); supervision (lead); writing – review and editing (lead).

## CONFLICT OF INTEREST STATEMENT

The authors have no relevant financial or nonfinancial interests to disclose.

## Supporting information


Data S1.

## Data Availability

The authors confirm that the data supporting the findings of this study are available within the article and its supplementary materials; first‐hand data supporting the results of this study are available from the corresponding author upon reasonable request.
