# Correction to “The Role of Heterotrophic Plasticity in Coral Response to Natural Low‐Light Environments”

**DOI:** 10.1002/ece3.72581

**Published:** 2025-12-10

Luo Y., Yu X., Huang L., Gan J., Lei X., Jiang L., Liu C., Sun Y., Cheng M., Zhang Y., Zhou G., Liu S., Lian J., and Huang, H. “The Role of Heterotrophic Plasticity in Coral Response to Natural Low‐Light Environments.” *Ecology and Evolution*. 14, (2024): e70278. https://doi.org/10.1002/ece3.70278.

In the funding information section, the text “Science and Technology Projects in Guangzhou, Grant/Award Number: E4D15C01;” was incorrect. This should have read “Guangzhou Science and Technology Plan Project, grant/award number: 2024A04J3951;”

We apologize for this error.

