# Supplementary material for: The role of heterotrophic plasticity in coral response to natural low‐light environments
Source: Ecol Evol. 2024 Sep 23;14(9):e70278. doi: 10.1002/ece3.70278 (PMC11420107; doi:10.1002/ece3.70278)
Supplement: Supplementary file 1 — Data S1. [file ECE3-14-e70278-s001.docx]

**Supplementary Material**


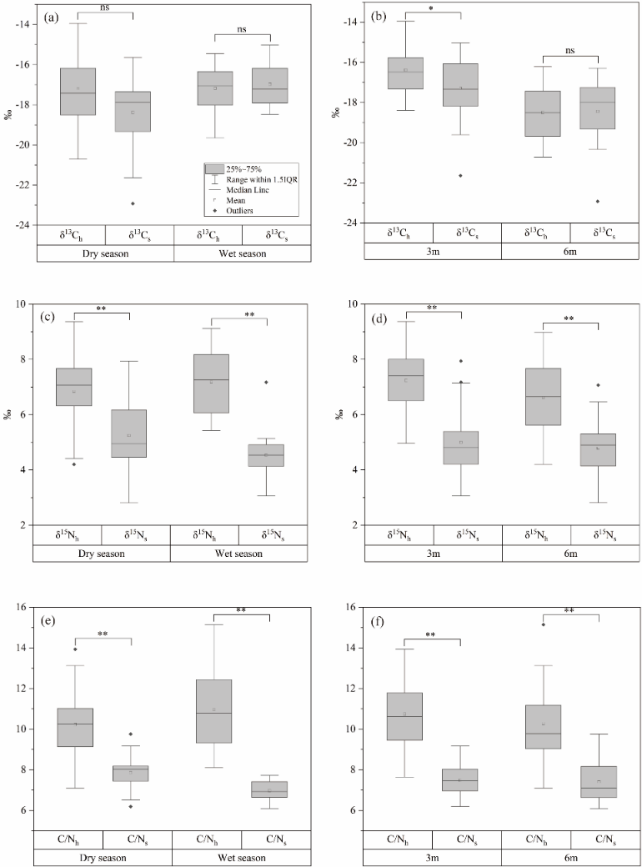


**Fig. S1.** Box plots of summary statistics averaged over all data for δ^13^C, δ^15^N, and C/N of coral hosts and symbionts at different seasons (a, c, e) and depths (b, d, f). Each box plot was calculated using n = 18 and n = 21 for the wet and dry seasons, respectively, while n = 24 and n = 15 for 3 m and 6 m, respectively. Box plots depict medians (central horizontal lines), interquartile ranges (boxes), 95% confidence intervals (whiskers), and outliers (solid diamonds). The asterisks denote significant differences (**p* < 0.05, ***p* < 0.01); ns: no significant differences.


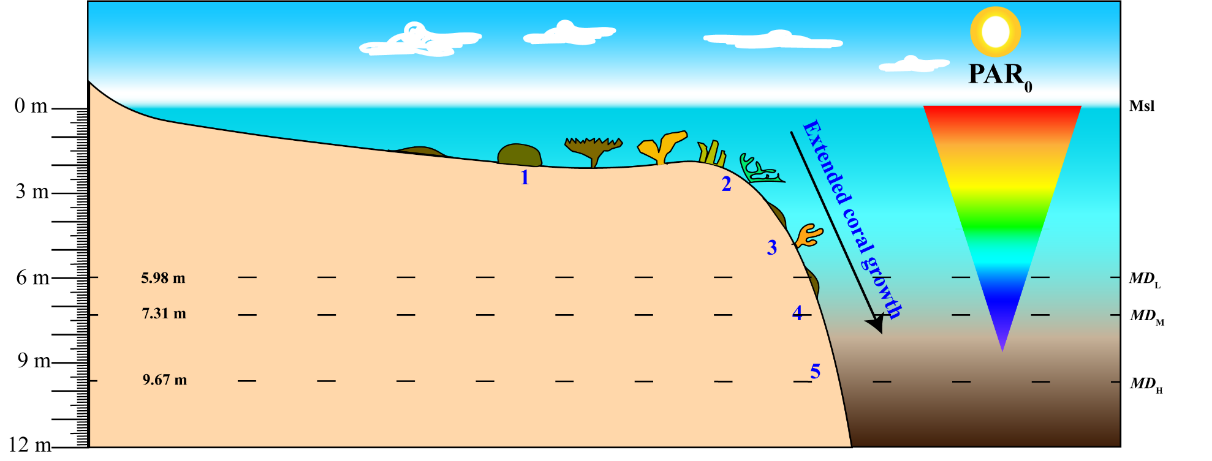


**Fig. S2.** The maximum depth distribution of coral *G. fascicularis* on the Luhuitou fringing reef. *MD*_H_: the high value of the maximum depth, *MD*_M_: the mean value of the maximum depth, *MD*_L_: the low value of the maximum depth, PAR_0_: surface photosynthetically active radiation, 1 = reef flat, 2 = reef crest, 3 = upper reef slope, 4 = lower reef slope, 5 = deep reef slope, Msl = mean sea level.

**Table S1.** Summary statistics for the Bayesian mixing models for global and depth during the wet (a-b) and dry (c-d) seasons produced via MixSIAR with trophic enrichment for heterotrophic resources (DIM, DOM, and POM) as shown in Table S1. Percentages represent the upper and lower credible intervals for the contribution of each source to the consumer, such that between 2.5% - 97.5% represent 95% of the variability in estimated source contribution to the consumer and 50% represents the median estimate. DIM = dissolved inorganic matter, DOM = dissolved organic matter, and POM = particulate organic matter.

| a) Wet season_ Global | Mean | SD | 2.5% | 5.0% | 25.0% | 50.0% | 75.0% | 95.0% | 97.5% |
| --- | --- | --- | --- | --- | --- | --- | --- | --- | --- |
| Epsilon.1 | 0.768 | 0.501 | 0.300 | 0.334 | 0.480 | 0.644 | 0.905 | 1.576 | 1.873 |
| Epsilon.2 | 1.363 | 0.873 | 0.430 | 0.503 | 0.796 | 1.142 | 1.644 | 2.997 | 3.663 |
| Global.DIM | 0.459 | 0.056 | 0.344 | 0.366 | 0.425 | 0.461 | 0.496 | 0.545 | 0.562 |
| Global.DOM | 0.013 | 0.015 | 0.000 | 0.000 | 0.003 | 0.008 | 0.018 | 0.043 | 0.054 |
| Global.POM | 0.528 | 0.058 | 0.415 | 0.435 | 0.490 | 0.527 | 0.564 | 0.623 | 0.648 |

| b) Wet season_ Depth | Mean | | SD | | 2.5% | | 5.0% | | 25.0% | | 50.0% | | 75.0% | | 95.0% | | 97.5% |
| --- | --- | --- | --- | --- | --- | --- | --- | --- | --- | --- | --- | --- | --- | --- | --- | --- | --- |
| Epsilon.1 | 0.731 | | 0.480 | | 0.260 | | 0.303 | | 0.452 | | 0.608 | | 0.855 | | 1.510 | | 1.979 |
| Epsilon.2 | 1.423 | | 0.941 | | 0.435 | | 0.499 | | 0.824 | | 1.176 | | 1.712 | | 3.186 | | 3.857 |
| p.3 m.DIM | 0.477 | | 0.063 | | 0.344 | | 0.371 | | 0.438 | | 0.479 | | 0.520 | | 0.573 | | 0.591 |
| p.6 m.DIM | 0.415 | | 0.075 | | 0.267 | | 0.289 | | 0.365 | | 0.416 | | 0.465 | | 0.536 | | 0.564 |
| p.3 m.DOM | 0.010 | | 0.012 | | 0.000 | | 0.000 | | 0.002 | | 0.005 | | 0.012 | | 0.034 | | 0.045 |
| p.6 m.DOM | 0.012 | | 0.018 | | 0.000 | | 0.000 | | 0.001 | | 0.005 | | 0.014 | | 0.050 | | 0.067 |
| p.3 m.POM | 0.513 | | 0.064 | | 0.394 | | 0.413 | | 0.470 | | 0.512 | | 0.554 | | 0.620 | | 0.648 |
| p.6 m.POM | 0.573 | | 0.078 | | 0.415 | | 0.445 | | 0.521 | | 0.574 | | 0.625 | | 0.702 | | 0.727 |
|  | | | | | | | | | | | | | | | | | |
| c) Dry season_ Global | Mean | SD | | 2.5% | | 5.0% | | 25.0% | | 50.0% | | 75.0% | | 95.0% | | 97.5% | |
| Epsilon.1 | 0.990 | 0.403 | | 0.472 | | 0.520 | | 0.717 | | 0.911 | | 1.165 | | 1.744 | | 2.050 | |
| Epsilon.2 | 14.356 | 3.472 | | 6.908 | | 8.241 | | 11.918 | | 14.657 | | 17.213 | | 19.409 | | 19.674 | |
| Global.DIM | 0.725 | 0.061 | | 0.618 | | 0.631 | | 0.683 | | 0.721 | | 0.765 | | 0.831 | | 0.854 | |
| Global.DOM | 0.193 | 0.096 | | 0.007 | | 0.023 | | 0.121 | | 0.205 | | 0.269 | | 0.332 | | 0.350 | |
| Global.POM | 0.082 | 0.079 | | 0.001 | | 0.002 | | 0.018 | | 0.054 | | 0.127 | | 0.250 | | 0.271 | |

| d) Dry season_ Depth | Mean | SD | 2.5% | 5.0% | 25.0% | 50.0% | 75.0% | 95.0% | 97.5% |
| --- | --- | --- | --- | --- | --- | --- | --- | --- | --- |
| Epsilon.1 | 0.308 | 0.159 | 0.125 | 0.140 | 0.203 | 0.270 | 0.368 | 0.598 | 0.716 |
| Epsilon.2 | 14.480 | 3.550 | 7.052 | 8.122 | 11.911 | 14.932 | 17.397 | 19.454 | 19.666 |
| p.3 m.DIM | 0.812 | 0.043 | 0.725 | 0.740 | 0.787 | 0.815 | 0.841 | 0.878 | 0.891 |
| p.6 m.DIM | 0.582 | 0.066 | 0.465 | 0.482 | 0.534 | 0.578 | 0.623 | 0.696 | 0.725 |
| p.3 m.DOM | 0.118 | 0.062 | 0.008 | 0.016 | 0.070 | 0.119 | 0.163 | 0.220 | 0.235 |
| p.6 m.DOM | 0.262 | 0.142 | 0.014 | 0.027 | 0.143 | 0.272 | 0.381 | 0.471 | 0.489 |
| p.3 m.POM | 0.070 | 0.059 | 0.003 | 0.005 | 0.021 | 0.053 | 0.106 | 0.184 | 0.209 |
| p.6 m.POM | 0.156 | 0.138 | 0.003 | 0.006 | 0.036 | 0.113 | 0.259 | 0.420 | 0.455 |

**Table S2.** Summary of mean values of δ^13^C, δ^15^N, and C/N for some coral species (hosts and symbionts) reported in the literature.

| Coral species | Depth (m) | δ^13^C_h_  (‰) | δ^13^C_s_  (‰) | δ^13^C_h-s_  (‰) | δ^15^N_h_  (‰) | δ^15^N_s_  (‰) | δ^15^N_h-s_  (‰) | C:N_h_  (mole mole^−1^) | C:N_s_  (mole mole^−1^) | C/N_h-s_  (mole mole^−1^) | References |
| --- | --- | --- | --- | --- | --- | --- | --- | --- | --- | --- | --- |
| *Galaxea fascicularis* | 3 | −16.38 ± 1.22 | −17.29 ± 1.54 | 0.91 ± 1.98 | 7.24 ± 1.23 | 5.00 ± 1.24 | 2.24 ± 1.50 | 10.75 ± 1.69 | 7.48 ± 0.71 | 3.27 ± 1.74 | This study |
|  | 6 | −18.50 ± 1.30 | −18.44 ± 1.71 | −0.05 ± 1.61 | 6.62 ± 1.33 | 4.79 ± 1.18 | 1.83 ± 2.24 | 10.28 ± 2.06 | 7.41 ± 0.96 | 2.87 ± 2.22 |  |
| *G. fascicularis* | 10 | −15.5 ± 1.2 | −15.0 ± 1.3 | N. | 5.4 ± 0.6 | 4.3 ± 0.5 | N. | 6.3 ± 0.6 | 7.9 ± 1.0 | N. | Radice et al. (2019) |
|  | 30 | −17.6 ± 0.8 | −17.2 ± 1.0 | N. | 5.6 ± 0.4 | 4.9 ± 0.7 | N. | 6.1 ± 0.5 | 8.1 ± 1.3 | N. |  |
| *Pachyseris speciosa* | 10 | −15.2 ± 1.4 | −14.0 ± 2.1 | N. | 5.2 ± 0.5 | 5.3 ± 0.5 | N. | 6.4 ± 0.4 | 9.7 ± 1.5 | N. |  |
|  | 30 | −15.1 ± 1.0 | −14.1 ± 1.6 | N. | 5.4 ± 0.5 | 5.7 ± 0.6 | N. | 6.4 ± 0.2 | 9.8 ± 1.6 | N. |  |
| *Pocillopora verrucosa* | 10 | −17.4 ± 1.1 | −16.3 ± 1.3 | N. | 5.8 ± 0.3 | 5.4 ± 0.6 | N. | 5.7 ± 0.3 | 7.1 ± 1.1 | N. |  |
|  | 30 | −18.0 ± 0.8 | −17.0 ± 0.9 | N. | 5.4 ± 0.4 | 5.2 ± 0.6 | N. | 6.0 ± 0.4 | 6.8 ± 0.9 | N. |  |
| *Porites lutea* | 4-6 | −14.28 ± 0.94 | −12.80 ± 0.72 | −1.48 ± 1.12 | N. | N. | N. | N. | N. | N. | Xu et al. (2020) |
| *Favia palauensis* |  | −13.86 ± 0.69 | −12.85 ± 0.61 | −1.00 ± 0.86 | N. | N. | N. | N. | N. | N. |  |
| *Pavona decussata* |  | −13.85 ± 0.66 | −13.27 ± 0.75 | −0.58 ± 0.90 | N. | N. | N. | N. | N. | N. |  |
| *Favites abdita* |  | −14.62 ± 0.59 | −13.02 ± 0.52 | −1.60 ± 0.65 | N. | N. | N. | N. | N. | N. |  |
| *F. palauensis* |  | −14.38 ± 0.41 | −13.05 ± 0.47 | −1.33 ± 0.48 | N. | N. | N. | N. | N. | N. |  |
| *F. palauensis* | 2-4 | −13.27 ± 0.95 | −12.52 ± 1.09 | −0.75 ± 1.06 | N. | N. | N. | N. | N. | N. | Xu et al. (2021) |
|  |  | −15.34 ± 1.1 | −13.27 ± 1.42 | −2.08 ± 1.37 | N. | N. | N. | N. | N. | N. |  |
|  |  | −15.63 ± 1.16 | −13.45 ± 1.06 | −2.18 ± 1.36 | N. | N. | N. | N. | N. | N. |  |
| *Madracis mirabilis* | 1 | −18.98 | −18.37 | N. | 3.90 | 3.54 | N. | N. | N. | N. | Muscatine, Porter, & Kaplan. (1989) |
|  | 10 | −17.74 | −16.79 | N. | 3.05 | 3.26 | N. | N. | N. | N. |  |
|  | 30 | −19.64 | −16.35 | N. | 1.84 | 2.64 | N. | N. | N. | N. |  |
| *Acropora cervicornis* | 1 | N. | −13.89 | N. | 4.11 | 1.76 | N. | N. | N. | N. |  |
|  | 10 | −15.34 | −14.05 | N. | 1.86 | 1.68 | N. | N. | N. | N. |  |
|  | 30 | −16.02 | −16.02 | N. | 1.56 | 0.16 | N. | N. | N. | N. |  |
| *Agaricia agaricites* | 1 | −13.08 | −12.52 | N. | 3.02 | 1.64 | N. | N. | N. | N. |  |
|  | 10 | −15.63 | −13.91 | N. | N. | 1.85 | N. | N. | N. | N. |  |
|  | 30 | −15.49 | −14.47 | N. | 1.48 | 1.86 | N. | N. | N. | N. |  |
|  | 50 | −17.90 | −15.61 | N. | 1.54 | 0.30 | N. | N. | N. | N. |  |
| *A. palmata* | 1 | −15.11 | −14.80 | N. | N. | 1.76 | N. | N. | N. | N. |  |
|  | 10 | −15.19 | −14.72 | N. | 2.13 | 1.48 | N. | N. | N. | N. |  |
| *P. astreoides* | 1 | −11.76 | −10.21 | N. | 2.79 | 2.99 | N. | N. | N. | N. |  |
|  | 10 | N. | −11.82 | N. | 2.10 | 2.30 | N. | N. | N. | N. |  |
|  | 30 | −17.91 | −14.64 | N. | 2.04 | 1.74 | N. | N. | N. | N. |  |
| *Montastraea annularis* | 1 | −11.90 | −9.63 | N. | 3.32 | 3.00 | N. | N. | N. | N. |  |
|  | 10 | −13.63 | −13.87 | N. | 2.41 | 1.83 | N. | N. | N. | N. |  |
|  | 30 | −19.64 | −18.33 | N. | 0.23 | 2.21 | N. | N. | N. | N. |  |
|  | 50 | −19.27 | −15.58 | N. | 1.87 | −0.16 | N. | N. | N. | N. |  |
| *M. cavernosa* | 1 | −10.75 | −11.28 | N. | 2.96 | 0.95 | N. | N. | N. | N. |  |
|  | 10 | −13.49 | −14.04 | N. | 1.11 | 0.35 | N. | N. | N. | N. |  |
|  | 30 | −16.79 | −14.67 | N. | 1.16 | −2.15 | N. | N. | N. | N. |  |
|  | 50 | −22.42 | −14.29 | N. | 3.43 | −1.73 | N. | N. | N. | N. |  |
| *Eusmilia fastigiata* | 1 | N. | −15.22 | N. | 3.45 | 3.45 | N. | N. | N. | N. |  |
|  | 10 | −15.39 | −15.12 | N. | 2.52 | 2.18 | N. | N. | N. | N. |  |
|  | 30 | −19.48 | −19.21 | N. | 2.76 | 0.94 | N. | N. | N. | N. |  |
| *Dendrogyra cylindrus* | 10 | −15.50 | −14.94 | N. | 2.23 | 2.43 | N. | N. | N. | N. |  |
| *Stylophora pistitlata* | 1 | −13.97 | −13.22 | N. | N. | N. | N. | N. | N. | N. | Muscatine and Kaplan. (1994) |
|  | 10 | −15.43 | −13.29 | N. | N. | N. | N. | N. | N. | N. |  |
|  | 30 | −18.59 | −15.74 | N. | N. | N. | N. | N. | N. | N. |  |
| *Porites* spp. | 3 | N. | N. | N. | N. | 7.4 ± 0.7 | N. | N. | N. | N. | Wong, Duprey, & Baker. (2017) |
|  |  | N. | N. | N. | N. | 4.7 ± 0.4 | N. | N. | N. | N. |  |
| *Montipora capitata* | 0.5-5 | −14.09 ± 2.09 | −15.62 ± 1.83 | 1.53 ± 0.63 | 4.36 ± 0.49 | 4.27 ± 0.44 | 0.09 ± 0.50 | N. | N. | N. | Price, McLachlan, Jury, Toonen, & Grottoli. (2021) |
| *M. patula* |  | −15.66 ± 1.29 | −17.01 ± 1.05 | 1.35 ± 0.69 | 4.99 ± 0.73 | 3.76 ± 0.78 | 1.23 ± 0.66 | N. | N. | N. |  |
| *P. acuta* |  | −15.89 ± 1.06 | −16.63 ± 0.87 | 0.74 ± 1.00 | 5.42 ± 1.17 | 4.00 ± 1.16 | 1.42 ± 0.95 | N. | N. | N. |  |
| *P. meandrina* |  | −14.77 ± 1.29 | −14.95 ± 0.91 | 0.19 ± 0.76 | 4.53 ± 0.75 | 4.28 ± 0.74 | 0.25 ± 0.71 | N. | N. | N. |  |
| *P. compressa* |  | −15.09 ± 1.07 | −15.02 ± 1.04 | −0.07 ± 0.59 | 3.66 ± 0.62 | 4.35 ± 0.63 | −0.69 ± 0.92 | N. | N. | N. |  |
| *P. lobata* |  | −14.24 ± 1.17 | −14.17 ± 1.07 | −0.07 ± 0.55 | 3.81 ± 0.77 | 4.27 ± 0.64 | −0.46 ± 0.91 | N. | N. | N. |  |
| *P. evermanni* |  | −15.27 ± 1.43 | −15.14 ± 1.30 | −0.13 ± 0.47 | 2.43 ± 0.78 | 4.22 ± 0.77 | −1.79 ± 0.68 | N. | N. | N. |  |

N. denotes no data.

***References***

Muscatine, L., & Kaplan, I. R. (1994). Resource Partitioning by Reef Corals as Determined from Stable Isotope Composition II. ^15^N of Zooxanthellae and Animal Tissue versus Depth. *Pacific Science,* *48*(3), 304-312. <http://hdl.handle.net/10125/2240>

Muscatine, L., Porter, J. W., & Kaplan, I. R. (1989). Resource partitioning by reef corals as determined from stable isotope composition. *Marine Biology, 100*(2), 185-193. doi:10.1007/bf00391957

Price, J. T., McLachlan, R. H., Jury, C. P., Toonen, R. J., & Grottoli, A. G. (2021). Isotopic approaches to estimating the contribution of heterotrophic sources to Hawaiian corals. *Limnology and Oceanography, 66*(6), 2393-2407. doi:10.1002/lno.11760

Radice, V. Z., Hoegh‐Guldberg, O., Fry, B., Fox, M. D., Dove, S. G., & Dorrepaal, E. (2019). Upwelling as the major source of nitrogen for shallow and deep reef‐building corals across an oceanic atoll system. *Functional Ecology, 33*(6), 1120-1134. doi:10.1111/1365-2435.13314

Wong, C. W., Duprey, N. N., & Baker, D. M. (2017). New Insights on the Nitrogen Footprint of a Coastal Megalopolis from Coral-Hosted Symbiodinium delta(15)N. *Environ Sci Technol, 51*(4), 1981-1987. doi:10.1021/acs.est.6b03407

Xu, S., Yu, K., Zhang, Z., Chen, B., Qin, Z., Huang, X., . . . Wang, Y. (2020). Intergeneric Differences in Trophic Status of Scleractinian Corals From Weizhou Island, Northern South China Sea: Implication for Their Different Environmental Stress Tolerance. *Journal of Geophysical Research: Biogeosciences, 125*(5), e2019JG005451. doi:10.1029/2019jg005451

Xu, S., Zhang, Z., Yu, K., Huang, X., Chen, H., Qin, Z., & Liang, R. (2021). Spatial variations in the trophic status of Favia palauensis corals in the South China Sea: Insights into their different adaptabilities under contrasting environmental conditions. *Science China Earth Sciences, 64*(6), 839-852. doi:10.1007/s11430-020-9774-0
